# High-Efficiency Utilization of Waste Tobacco Stems to Synthesize Novel Biomass-Based Carbon Dots for Precise Detection of Tetracycline Antibiotic Residues

**DOI:** 10.3390/nano12183241

**Published:** 2022-09-18

**Authors:** Hui Yang, Yunlong Wei, Xiufang Yan, Chao Nie, Zhenchun Sun, Likai Hao, Xiankun Su

**Affiliations:** 1 State Key Laboratory of Environmental Geochemistry, Institute of Geochemistry, Chinese Academy of Sciences, Guiyang 550081, China; 2 Guizhou Academy of Tobacco Science, Guiyang 550081, China; 3 University of Chinese Academy of Sciences, Beijing 100049, China; 4 School of Food & Biological Engineering, Key Laboratory for Agricultural Products Processing of Anhui Province, Hefei University of Technology, Hefei 230009, China; 5 Key Laboratory of Tobacco Quality Research of Guizhou Province, College of Tobacco Science, Guizhou University, Guiyang 550025, China

**Keywords:** waste tobacco stems, biomass-based C−dots, tetracycline antibiotics, inner filter effect, environmental pollution traceability analysis

## Abstract

Recycling waste biomass into valuable products (e.g., nanomaterials) is of considerable theoretical and practical significance to achieve future sustainable development. Here, we propose a one-pot hydrothermal synthesis route to convert waste tobacco stems into biomass-based N, S-codoped carbon dots (C−dots) with the assistance of carbon black. Unlike most of the previously reported luminescent C−dots, these biomass-based C−dots showed a satisfactory stability, as well as an excitation-independent fluorescence emission at ~520 nm. Furthermore, they demonstrated a pH-dependent fluorescence emission ability, offering a scaffold to design pH-responsive assays. Moreover, these as-synthesized biomass-based C−dots exhibited a fluorescence response ability toward tetracycline antibiotics (TCs, e.g., TC, CTC, and OTC) through the inner filter effect (IFE), thereby allowing for the establishment a smart analytical platform to sensitively and selectively monitor residual TCs in real environmental water samples. In this study, we explored the conversion of waste tobacco stems into sustainable biomass-based C−dots to develop simple, efficient, label-free, reliable, low-cost, and eco-friendly analytical platforms for environmental pollution traceability analysis, which might provide a novel insight to resolve the ecological and environmental issues derived from waste tobacco stems.

## 1. Introduction

Recently, making full use of waste biomass to produce valuable products has become an important way to achieve green and sustainable development, in line with the global consensus [1]. Driven by this motivation, a variety of biomass-derived nanomaterials have emerged and are widely used in biological/chemical sensing [2,3,4,5,6,7]. Among them, biomass-based carbon dots (C−dots), a promising fluorescent carbon-based nanomaterial, have been widely developed for applications such as environmental pollution traceability analysis, food safety assessment, bioimaging, fluorescent ink, photocatalysis, etc., owing to their unique optical properties, low cost, water solubility, high stability, biocompatibility, and eco-friendliness [8,9,10]. Until now, the reported biobased C−dots have been primarily derived from the biomass-related precursors of tree leaves [11,12], corn stalk shells [13,14], peanut shells [15], coffee [16], capsicum [17], and watermelon peel through various thermal synthesis techniques [18], but little attention has been focused on employing waste tobacco stems as a biomass−related precursor to synthesize efficient and green biomass-based C−dots. A considerable amount of waste tobacco stems are produced every year in the production of cigarettes and cigars [19]. Therefore, the reasonable recycling and utilization of these waste tobacco stems to develop new C−dots−based assays is an important way to turn waste into treasure, which is of considerable practical and theoretical significance to achieve sustainable green development.

In recent decades, broad-spectrum, antibacterial, feature-driven disease-prevention efficacy has resulted in the overuse of antibiotics in clinical and poultry farming fields, resulting in increasingly prominent environmental pollution and food safety problems and seriously threating public health [20,21,22]. Among a wide variety of antibiotics, tetracycline antibiotics (TCs), including tetracycline (TC), oxytetracycline (OTC), and aureomycin (CTC), are widely employed as a food additive in animal feeding, owing to their low cost and bactericidal ability [23,24]. It has been reported that TC antibiotics are capable of binding with the ribosomes of both Gram−positive and Gram-negative bacteria, blocking protein synthesis to trigger bacterial death [25]. Nevertheless, the overuse of TCs has inevitably led to the presence of TC residues in environmental water and soil, which could be further accumulated in the human body by means of food chains [26,27]. Accordingly, long−term accumulated TCs could cause a series of side effects on human health, such as allergic symptoms, hepatotoxicity, skeletal dysplasia, chronic toxicity, etc. [28]. More seriously, they could lead to the emergence of drug−resistant bacteria, seriously threatening public safety [29]. Therefore, it is of considerable importance to develop an accurate, reliable, simple, economic, eco−friendly, and label-free assay to monitor TC residues for environmental pollution traceability and food safety risk assessment [30].

**Figure 1:**
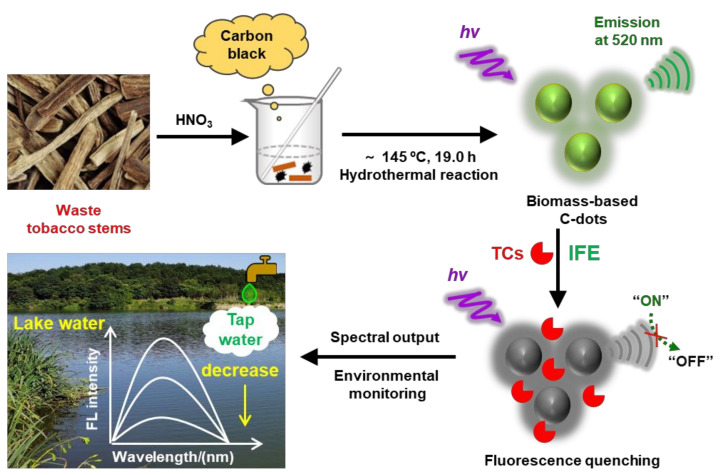
Illustration of the synthesis procedure for waste tobacco stems biomass−based C−dots and their fluorescence response mechanism toward TC residues.

Inspired by this need, in the present study, we focused on utilizing carbon-black-assisted waste tobacco stems as biomass-related precursors to greenly synthesize efficient biomass-based C−dots for the development of a precise, reliable, simple, low-cost, environmentally friendly, and label-free assay for TC residue monitoring. We adopted a one-pot hydrothermal synthesis method to synthesize novel yellow−green fluorescence biomass−based C−dots with a unique excitation-independent fluorescence emission at ~520 nm. Through characterization and analysis, these as−synthesized biomass-based C−dots were codoped with N and S elements, with a homogeneous surface state of the sp2 clusters. Owing to the inner filter effect, TCs could selectively trigger the remarkable fluorescence of these as-synthesized C−dots, demonstrating a TC−concentration−dependent decrease in fluorescence, making them suitable for a label−ree, sensitive, selective, and eco-friendly fluorescence assay for TC residue monitoring in environmental water samples (Figure 1). The corresponding limit of detection (LOD) for TC was 1.328 nM in a linear range from 0.004 to 100.0 μM. Likewise, the LOD for CTC was 3.234 nM, with a linear range of 0.011−100.0 μM. Furthermore, the LOD for OTC was 9.881 nM, with a linear range of 0.033−200.0 μM. This study shows the considerable application prospects of waste tobacco stems as biomass precursors to synthesize green biomass-based C−dots for the development of efficient, low−cost, and eco−friendly environmental pollution traceability and food safety analysis methods.

## 2. Materials and Methods

Reagents and Chemicals. Waste tobacco stems (2021, Guizhou, China) were provided by Guizhou Academy of Tobacco Science. Carbon black, nitric acid (HNO_3_), phosphoric acid (H_3_PO_4_), boric acid (H_3_BO_3_), acetic acid (HAc), sodium hydroxide (NaOH), hydrochloric acid (HCl), barium chloride (BaCl_2_), ferric chloride (FeCl_3_), strontium chloride (SrCl_2_), magnesium sulfate (MgSO_4_), calcium sulfate (CaSO_4_), cobalt chloride (CoCl_2_), tin chloride (SnCl_2_), lead acetate ((CH_3_COO)_2_Pb), copper sulfate (CuSO_4_), manganese sulfate (MnSO_4_), zinc sulfate (ZnSO4), mercury sulfate (HgSO_4_), platinum chloride (PtCl_4_), palladium nitrate (Pd (NO_3_)_2_), aluminum chloride (AlCl_3_), vanadium chloride (VCl_3_), chromium chloride (VCl_2_), nickel chloride (NiCl_2_), cadmium chloride (CdCl_2_), silver nitrate (AgNO_3_), tetracycline hydrochloride (TC), oxytetracycline hydrochloride (OTC), chlortetracycline hydrochloride (CTC), penicillin G sodium (PenG), cephalothin sodium (CF), DL-penicillin (DL−PEN), streptomycin sulfate (STR), kanamycin sulfate (KAN), citric acid (CA), glycine (Gly), homocysteine (L−Hcy), phenylalanine (L−PHE), lysine (L−Lys), cysteine (L−Cys), valine (L−Val), proline (L−Pro), tyrosine (L−Tyr), glutathione (GSH), ascorbic acid (AA), ethanol, acetone, acetonitrile, dimethyl sulfoxide (DMSO), and tetrahydrofuran (THF) were purchased from Aladdin Biochemical Technology Co., Ltd. (Shanghai, China). Britton−Robinson (B−R) buffer with a pH range of 2.0−12.0 was obtained by adding varying volumes of NaOH (0.2 M) to 100.0 mL of a mixture of H_3_PO_4_, H_3_BO_3_, and HAc (all at 0.04 M concentrations). All chemicals were used untreated. D.I. water (H_2_O) was used throughout the experiment (Milli-Q, Meck, Darmstadt, Germany).

Apparatus. Fluorescence spectroscopy (F-7000, Hitachi, Tokyo, Japan) and UV-visible spectrophotometry (Carry 50, Varian, Santa Clara, CA, USA) were used to determine all fluorescence and absorption spectra in this work, respectively. High-resolution transmission electron microscopy (TEM) (Tecnai G2 F20 S-Twin (200 kV), FEI, Hillsboro, OR, USA) was used to observe the morphology, size, and lattice structure of the biomass-based C−dots. Fourier transform infrared (FT−IR) spectroscopy (Nicolet IS10, Thermo Nicolet, Waltham, MA, USA), X-ray photoelectron spectroscopy (Escalab 250Xi, Thermo Scientific, Waltham, MA, USA), Raman spectrometry (inVia, RENISHAW, Wotton-under-Edge, UK), X-ray diffractometry (D8 ADVANCE, BRUCKER, Bremen, Germany), and hydrogen spectrum nuclear magnetism (AVANCE III, BRUCKER, Bremen, Germany) were used to characterize the composition and surface functional groups of the biomass-based C−dots. Transient fluorescence spectrometry (JY HORIBA Fluorolog-3, Jobin Yvon Inc., Edison, NJ, USA) was used to determine the fluorescence decay curves. Biomass-based C−dots powder was obtained in a freeze dryer (VaCo 5, Zirbus, Bad Grund, Germany). A Zetasizer (Nano−ZS, Malvern, UK) was applied to measure the surface zeta potential. pH was measured in an aqueous solution on a Mettler Toledo pH meter (S400-B, Mettler Toledo, Greifense, Switzerland).

### 2.1. Synthesis of Biomass-Based C−Dots

First, 0.5 g of waste tobacco stems and 0.5 g of carbon black were dissolved in 100.0 mL of 14.5 M HNO_3_. The mixture solution was then transferred into a 250.0 mL round-bottomed flask and heated at 145.0 °C for reflux reaction for 19.0 h. When the reactor was cooled to ambient temperature, the insoluble precipitate was removed by centrifugation (4000.0 rpm, 30.0 min), and the supernatant was retained. Then, the solution pH was adjusted to 7.0 with NaOH solution and filtered through a 0.22 μm filter membrane. Finally, after 5 days of dialysis in a dialysis bag (M_W_ = 3500 Da), the water was changed every 6.0 h and freeze−dried to obtain a brown C−dots solid powder, which was dried at room temperature and stored for later use.

### 2.2. Detection of TCs with a Biomass-Based C−Dots Assay

Taking detection of TC as an example, varying amounts of TC were added to the C−dots solution and mixed evenly in a quartz cuvette. After incubation at room temperature for 10.0 min, the fluorescence emission spectra of the mixture were recorded on a fluorescence spectrophotometer. The fluorescence intensity at 520 nm was monitored at the excitation wavelength of 360 nm (Ex/Em: 5/5 nm slit width), and the relationship between the intensity of 520 nm and TC concentration (range 0.0 to 200.0 μM) was systematically studied. In addition, the color changes of the fluorescence mixture were observed under a 365 nm UV lamp and photographed with a camera. CTC and OTC testing procedures are consistent with the above procedures.

### 2.3. Selectivity and Interference Experiments

To explore the selectivity of biomass-based C−dots for TCs, their fluorescence response to common interfering substances in environmental water samples was measured, including common metal ions (Hg^2+^, Al^3+^, Ba^2+^, Ca^2+^, Cd^2+^, Co^2+^, Cu^2+^, Fe^3+^, Mg^2+^, Mn^2+^, Ni^2+^, Zn^2+^, V^3+^, V^2+^, Sr^2+^, Sn^2+^, Pt^4+^, Pd^2+^, Pb^2+^, and Ag^+^), anions (Cl^−^, I^−^, NO_3_^−^, PO_4_^3−^, and SO_4_^2−^), representative amino acids (Gly, L−Hcy, L−Phe, L−Lys, and L−Cys), GSH, and AA. Some antibiotics (PenG, CF, DL−PEN, STR, and KAN) were also selected to demonstrate the specificity of the assay. In addition, interference tests for TCs and mixtures of coexisting substances were performed under the same conditions.

The process was as follows: 1000.0 μL C−dots (100.0 μg/mL) and 1000.0 μL BR (40.0 mM, pH = 5.0) were pipetted into a 2.0 mL EP tube. Subsequently, 1000.0 μL TC (or OTC or CTC) was added to the previously prepared C−dots, and vortex mixing was performed. Each of the above mixtures was reacted under optimized conditions of optimal C-dot concentration, temperature, pH, and time; specifically, TC (C−dots = 25.0 μg/mL, pH = 5.0, 20.0 °C, 10.0 min), OTC (C−dots = 10.0 μg/mL, pH = 4.0, 20.0 °C, 10.0 min), and CTC (C−dots = 25.0 μg/mL, pH = 4.0, 50.0 °C, 10.0 min).

### 2.4. Real Sample Analysis

Lake water and tap water were randomly taken from the Yueshan Lake Park and the laboratory faucet (Guiyang, China), respectively. All original samples were pretreated, centrifuged (4000.0 rpm, 30.0 min), and filtered through a 0.22 μm filter to remove the likely suspended particles. Then, a series of lake water and tap water samples containing varying concentrations of TCs (TC, CTC, or OTC) were prepared by spiking varying volumes of TC reserve solution (1.0 mM). Next, these real samples were added to an equal-volume solution of biomass-based C−dots. The solution was thoroughly mixed and incubated for 10.0/20.0 min. Finally, the emission spectra (λex = 360 nm) were recorded.

## 3. Results and Discussions

### 3.1. Synthesis and Characterization of Biomass−Based C−Dots

**Figure 2:**
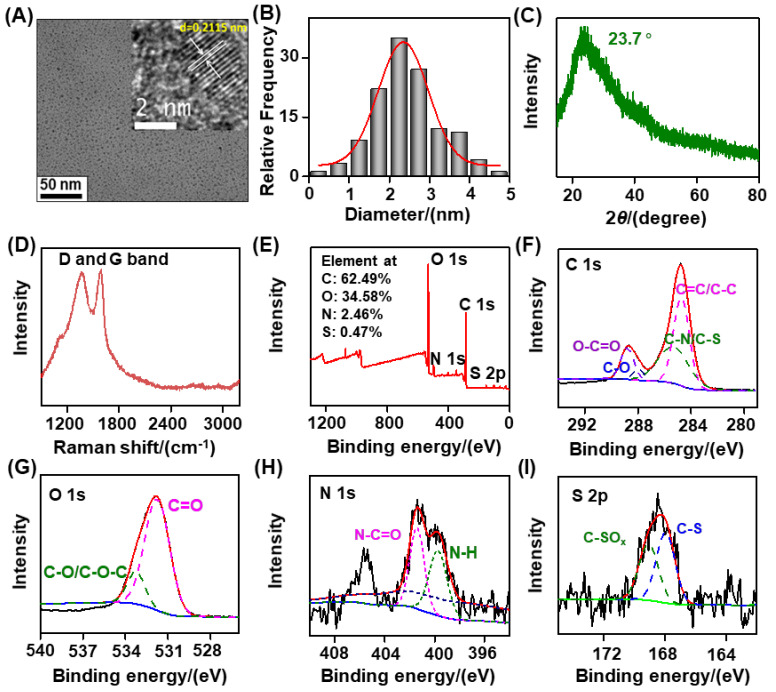
Characterization of the synthesized biomass−based C−dots. (**A**) TEM image, (**B**) size distribution, (**C**) XRD pattern, (**D**) Raman spectrum, (**E**) XPS survey, (**F**) C 1s, (**G**) O 1s, (**H**) N 1s, and (**I**) S 2p high−resolution spectra of the biomass-based C−dots. Inset of Figure 2A is the HRTEM image of the biomass−based C−dots.

Common methods for synthesizing C−dots include hydrothermal, microwave, and chemical oxidation routes. Hydrothermal synthesis is one of the most commonly used methods to prepare C−dots, usually referring to the process of carbonization and pyrolysis of biomass materials to form C−dots in a closed hydrothermal reaction kettle under high temperature and pressure. As an effective, simple, and conventional method, chemical oxidation is beneficial for the large−scale synthesis of biomass C−dots. In the synthesis process, the use of chemical oxidants (such as H_2_O_2_ and H_2_SO_4_/HNO_3_) can promote the carbonization of biomass materials to prepare biomass C−dots. According to previous reports, hydrothermal and chemical oxidant treatment−triggered intra- and intermolecular dehydration/decomposition is the classical synthesis method for biomass-based C−dots [31,32,33]. Accordingly, we attempted to adopt a chemical oxidation method to synthesize residual TC stimulus−responsive, biomass-based C−dots by employing carbon-black-assisted waste tobacco stems as biomass−related precursors. Transmission electron microscopy (TEM) imaging clearly revealed that these as−synthesized biomass-based C−dots were well−dispersed, with a sphere-like structure and an average dimeter of ~2.8 nm (Figure 2A,B). The high−resolution TEM (HRTEM) imaging further illustrated that these as-synthesized biomass-based C−dots exhibited a lattice spacing of ~0.2115 nm (inset of Figure 2A), which was less than that of natural graphite (~0.34 nm), implying the existence of small graphitic crystals [34]. Furthermore, a very sharp and strong diffraction peak of about 26° was observed near 2*θ*, that is, the diffraction peak of the graphite (002) plane, indicating that the spatial arrangement of the pure graphite microwafer layer was very regular. After graphite is oxidized, the diffraction peak of the graphite (002) surface is very small, but a strong diffraction peak of approximately 10.6° appears near 2*θ*, that is, the diffraction peak of the graphite oxide (001) surface, indicating that the crystal form of graphite was destroyed, and a new crystal structure was formed. When graphite oxide is reduced to graphene, the diffraction peak of graphene of approximately 23° appears at 2*θ*, which is similar to the diffraction peak of graphite, although it becomes wider, and the intensity weakens because after reduction, the graphite lamellar size is reduced, the integrity of the crystal structure decreases, and disorder increases. Due to the disorder of carbon atoms and the existence of C−dots with a large number of edge and surface defects, XRD patterns usually show that biomass-based C−dots have amorphous properties and generally have wide diffraction peaks in the range of 2*θ*=20°−25° [35]. However, if the prepared biomass C−dots have good crystallinity, the diffraction peaks of crystal planes (002), (100), (004), and (110) can also be observed from the spectrogram [36]. Furthermore, there was an obvious peak at 2*θ* = 23.7° in the XRD pattern of these biomass-based C−dots (Figure 2C), indicating a graphene structure with a surface rich in oxygen-containing groups. Therefore, it is speculated that the surface of the biomass-based C−dots synthesized in this study may contain functional groups similar to graphene [37]. Moreover, the Raman peak at ~1372 cm^−1^ was ascribed to the disordered sp3 carbon−originated D band, and the other Raman peak at ~ 1593 cm^−1^ was the result of the graphitic sp2 carbon-formed G band. The comparable intensities of the D band and G band illustrate that these biomass-based C−dots were composed of many structural defects (Figure 2D) [38]. XPS analysis revealed that the as-synthesized C−dots mainly contained C, N, O, and S elements (Figure 2E). The C 1s high-resolution spectrum showed four peaks at ~284.7 eV, ~285.4 eV, ~287.9 eV, and ~288.8 eV, which were attributed to the groups of C=C/C-C, C-N/C-S, C-O, and O−C=O bonds, respectively (Figure 2F). Furthermore, the O 1s high-resolution spectrum exhibited two main peaks at ~531.8 eV and ~533.3 eV, which were derived from the C=O and C−O/C−O−C bonds, respectively (Figure 2G) [39]. The two main peaks at ~399.7 eV and ~401.4 eV in the N 1s high-resolution spectrum corresponded to the N−H and N−C=O bonds, respectively (Figure 2H). Apart from these, the two main peaks at 168.0 eV and 169.1 eV in the S 2p high-resolution spectrum were the result of C−S and C−SOx bonds, respectively (Figure 2I) [40]. According to FT-IR analysis, the characteristic absorption peak at ~3425 cm^−1^ was attributed to the stretching vibration of O-H/N-H, and the characteristic absorption peak at ~1710 cm^−1^ corresponded to the stretching vibration of C=O or C=C. The absorption peak at ~1419 cm^−1^ was related to C-H bending vibration, and the absorption peak at ~ 1236 cm^−1^ was related to C−O bending vibration. The characteristic absorption at ~1033 cm^−1^ implied C−S or S−O bonds inside the biomass-based C−dots (Figure S1) [41,42], in agreement with the XPS analysis. According to these analyses, the as-synthesized waste tobacco biomass−based C−dots contained rich functional groups, such as −COOH, −OH, −NH_2_, and C=O in their surface. Notably, the negative surface zeta potential (−13.6 ± 1.7 mV) indicated that the −COOH content was probably richer than that of −NH_2_ on the surface of the biomass-based C−dots (Figure S2).

### 3.2. Optical Features of the Biomass-Based C−Dots

**Figure 3:**
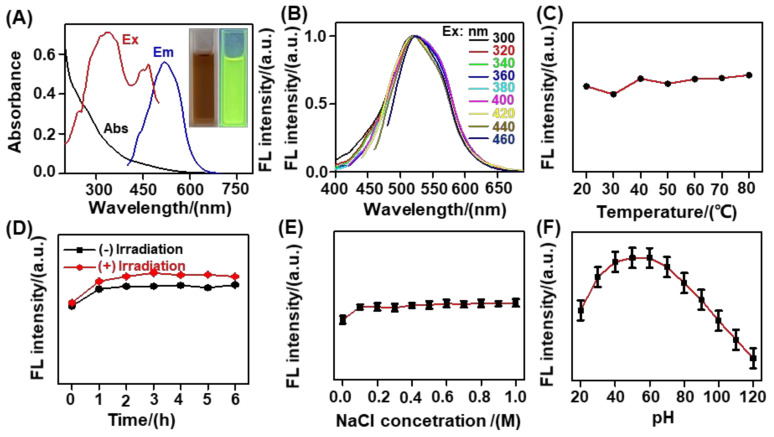
Optical features of biomass−based C−dots. (**A**) Absorption, excitation, and fluorescence emission spectra of biomass−based C−dots in D.I. water. Inset is a photograph and fluorescence image in D.I. water under a 365 nm UV lamp. (**B**) Fluorescence emission spectra of biomass−based C−dots in D.I. water upon excitation at varying wavelengths. (**C**) Normalized fluorescence intensity of biomass-based C−dots incubated at varying temperatures in D.I. water. (**D**) Normalized fluorescence intensities of biomass-based C−dots in D.I. water without (−) and with (+) LED irradiation for varying times. (**E**) Normalized fluorescence intensity of biomass−based C−dots in varying concentrations of NaCl solution. (**F**) Normalized fluorescence intensity of biomass-based C−dots in BR buffer at varying pH levels.

As shown in Figure 3A, there was a weak peak at ~265 nm in the absorption spectrum of the biomass−based C−dots, probably attributable to the π−π* transition of C=C and the n−π* transition of C=O. The biomass-based C−dots solution was yellow−brown, emitting yellow−green fluorescence under excitation with a 365 nm UV lamp. As shown in Figure 3B, upon excitation in the range of 300 to 460 nm, the biomass-based C−dots exhibited an excitation-independent fluorescence emission at ~520 nm, which was differed from that of general carbon-based fluorescence nanomaterials. Most carbon dots are dependent on fluorescence emission on the excitation wavelength, that is, with increased excitation wavelength, the emission spectrum of C−dots is redshifted, accompanied by a change in fluorescence intensity. The cause of this phenomenon may be the uneven particle size distribution of C−dots or could be related to the varying number and types of luminescent sites that can be excited during the change of excitation wavelength [43]. With respect to the excitation-independent emission of C−dots, some studies show that such properties of C−dots may be related to their surface oxidation state. C−dots with a high degree of oxidation contain more defects, so the fluorescence emission wavelength is dependent on excitation and vice versa [44], in agreement with our experimental results. The degree of oxidation of biomass-based C−dots prepared by the oxidation method may be low, so the emission wavelength does not depend on the excitation wavelength. This atypical fact confirms that our biomass−based C−dots had a homogeneous surface state of sp2 clusters, which was conducive to effectively avoiding autofluorescence interference toward analytical sensitivity in real environmental traceability analysis [45]. Because the maximum fluorescence intensity of these biomass-based C−dots appeared at 360 nm, 360 nm was selected as the optimal excitation wavelength to carry out all subsequent fluorescence analyses.

In general, the optical stability of synthesized biomass-based C−dots are an important consideration in the design of a promising analytical platform. Bearing this in mind, the effects of the incubation temperature, LED light irradiation, and ionic strength on the fluorescence emission of these biomass-based C−dots were examined. As depicted in Figure 3C, the fluorescence intensity of the biomass-based C−dots at ~520 nm was insignificantly impacted by incubation temperatures in the range of 20.0−80.0 °C for more than 30.0 min in D.I. water. As shown in Figure 3D, the effect of LED light irradiation on the fluorescence emission of the biomass-based C−dots was also negligible, owing to the minimal change in fluorescence intensity at ~520 nm for 6.0 h of continuous LED irradiation. This finding indicates that the biomass-based C−dots had exhibited good photobleaching resistance. In addition, the ionic strength effect on the C−dots fluorescence emission was examined by adding NaCl. As shown in Figure 3E, almost no change in fluorescence intensity of the biomass-based C−dots occurred at ~520 nm after the addition of NaCl concentrations in the range of 0.0−1.0 M, revealing that the biomass-based C−dots exhibited resistance to the interference of ionic strength. We also noticed that the biomass-based C−dots showed a concentration-dependent fluorescence emission, with the maximum fluorescence emission intensity observed at 50.0 μg/mL (Figure S3). We found that the fluorescence of the biomass-based C−dots was stronger in THF than other solvents, such as H_2_O (D.I. water), DMSO, DMF, CH_3_COCH_3_, CH_3_CH_2_OH, and CH_3_CN (Figure S4). Using rhodamine 6G as a reference, the fluorescence quantum yield of the biomass-based C−dots was ~19.45% in THF (Note S1). As illustrated in Figure 3F, the fluorescence emission of the biomass-based C−dots exhibited a pH−responsive feature, with the strongest fluorescence intensity observed at pH = 6.0. This pH−responsive feature reminded us that pH influence should be considered in the design of C−dots-based assays.

### 3.3. Fluorescence Response of this Biomass−Based C−Dots toward TCs Residues

Having demonstrated the optical features of the biomass-based C−dots, we then exhibited their luminescence to develop an analytical platform for monitoring of residual TCs in aqueous solution. We first examined the effects of pH, C−dots concentration, incubation time, and temperature on the fluorescence response of the biomass-based C−dots toward TCs. Taking TC as an example, we observed that the maximum fluorescence quenching extent was occurred at pH = 5.0 (Figure S5A), indicating that the optimal pH for TC detection was 5.0. Furthermore, the maximum C-dot fluorescence quenching efficiency caused by TC occurred at a biomass−based C−dots concentration of 25.0 μg/mL (Figure S5B). Moreover, we also noticed that the TC-triggered maximum fluorescence reduction of the biomass-based C−dots occurred at the incubation temperature of 20.0 °C (Figure S5C) and an incubation time of 10.0 min (Figure S5D). According to these findings, we selected the following optimal experimental conditions for the analytical process of TC monitoring: pH = 5.0; concentration of biomass-based C−dots, 25.0 μg/mL; incubation temperature, 20.0 °C; and incubation time, 10.0 min. Similar to the optimized conditions for TC assay, we further determined the optimal detection conditions for CTC (Figure S6): pH = 4.0, concentration of biomass-based C−dots, 25.0 μg/mL; incubation temperature, 50.0 °C; and incubation time, 10.0 min for performing CTC assay. Additionally, the optimal conditions for OTC were determined as min (Figure S7): pH = 4.0; concentration of biomass-based C−dots, 10.0 μg/mL; incubation temperature, 20.0 °C; and incubation time, 10.0.

**Figure 4:**
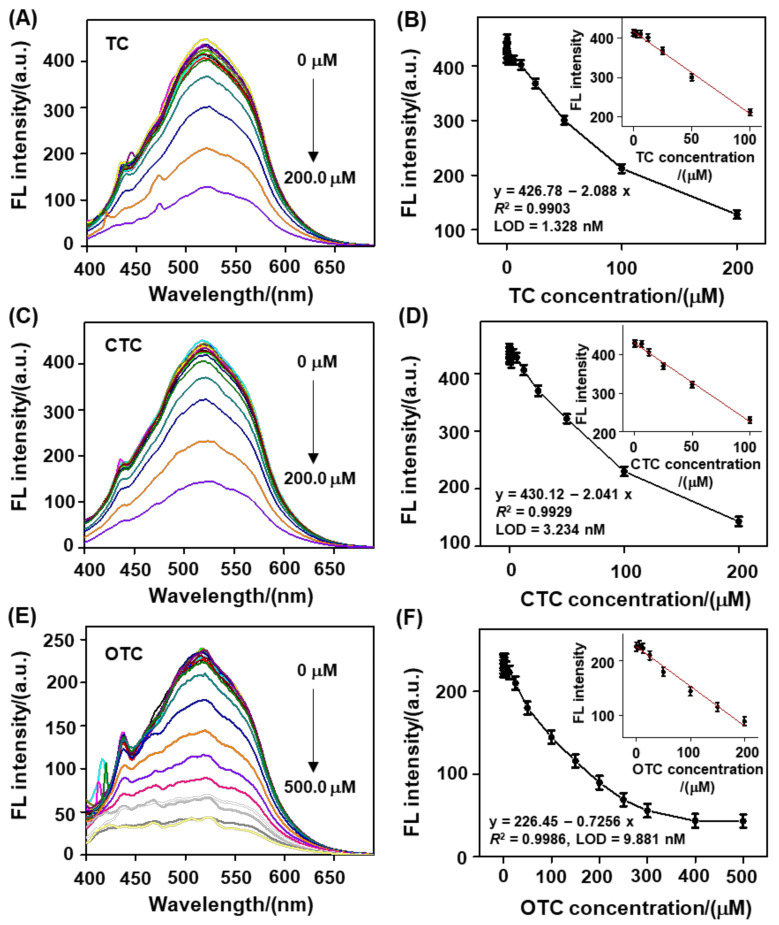
Fluorescence response of the biomass−based C−dots toward TCs in BR buffer. (**A**) Fluorescence emission spectra of the biomass−based C−dots (25.0 μg/mL) incubated with various concentrations of TC in the range of 0.0 to 200.0 μM. (**B**) C−dots florescence intensity plot at 520 nm versus TC concentration, as shown in Figure 4A. Inset: linearity of the C−dots florescence intensity at 520 nm versus CTC concentration in the range of 0.0−100.0 μM. Conditions: pH = 5.0; reaction time, 10.0 min; temperature, 20.0 °C. (**C**) Fluorescence emission spectra of the biomass-based C−dots (25.0 μg/mL) incubated with various concentrations of CTC in the range of 0.0 to 200.0 μM. (**D**) C−dots florescence intensity plot at 520 nm versus CTC concentration, as shown in Figure 4C. Inset: linearity of the C−dots florescence intensity at 520 nm versus CTC concentration in the range of 0.0−100.0 μM. Conditions: pH = 4.0; reaction time, 10.0 min; temperature, 50.0 °C. (**E**) Fluorescence emission spectra of the biomass−based C−dots (10.0 μg/mL) incubated with various concentrations of OTC in the range of 0.0 to 500.0 μM. (**F**) C-dot florescence intensity plot at 520 nm versus OTC concentration, as shown in Figure 4E. Inset: linearity of the C−dots florescence intensity at 520 nm versus OTC concentration in the range of 0.0−200.0 μM. Conditions: pH = 4.0; reaction time, 10.0 min; temperature, 20.0 °C.

Next, under the optimal conditions listed above, we observed that the fluorescence emission of the biomass-based C−dots displayed a TC concentration−dependent decrease in the range of 0.0 to 200.0 μM (Figure 4A). By plotting the fluorescence intensity (F) of the biomass-based C−dots at 520 nm versus TC concentration, a linear equation of *F/F*_0_ = 426.78−2.088 *C* (correlation coefficient *R*^2^ = 0.9903) was proposed with an LOD (3σ/slope, where σ corresponds to the standard deviation from eleven replicate measurements of the blank samples) of 1.328 nM and a linear range of 0.004−100.0 μM (Figure 4B). Likewise, we also noticed that there was a CTC/OTC concentration-dependent fluorescence decrease in the biomass-based C−dots at 520 nm in the range of 0.0–200.0 μM (Figure 4C) or 0.0−500.0 μM (Figure 4E). The corresponding linear equation for CTC was *F* = 430.12−2.041 *C* (*R*^2^ = 0.9929), with an LOD of 3.234 nM in the linear range of 0.011 to 100.0 μM (Figure 4D), and the linear equation for OTC was *F* = 226.45−0.7256 *C* (*R*^2^ = 0.9986), with an LOD of 9.881 nM in the linear range of 0.033 to 200.0 μM (Figure 4F). As compared with other reported fluorescence assays (Table S1), the biomass-based C-dot-driven analytical platform displayed a wider linear range, as well as comparable or even better sensitivity for monitoring of residual TCs. More significantly, these LODs were below the maximum allowable residue defined by China (100.0 mg/kg) and the European Union (225.0 nM) [46]. These results strongly imply that the proposed biomass-based C−dots are prominently sensitivity toward TCs, with practical potential for accurate traceability analysis of residual TCs in complex environmental matrices.

**Figure 5:**
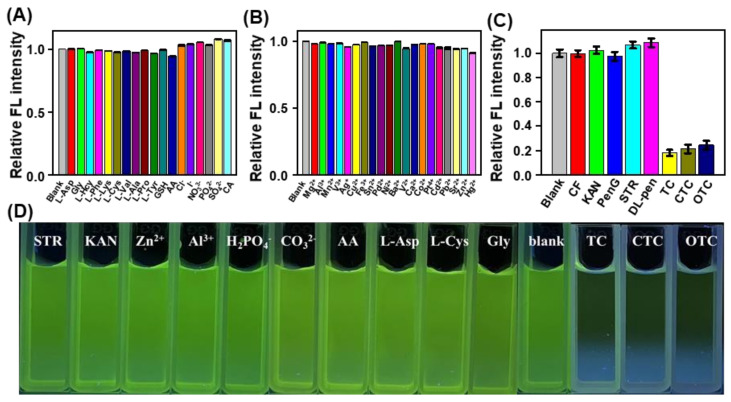
Influences of various interferences, such as (**A**) L−Asp, Gly, L−Hcy, L−Phe, L−Lys, L−Cys, L−Val, L−Ala, L−Pro, L−Tyr, GSH, AA, CA, Cl^−^, I^−^, NO_3_^−^, PO_4_^3−^, and SO_4_^2−^ (25.0 μM for each); (**B**) Mg^2+^, Al^3+^, Mn^2+^, V^3+^, Ag^+^, Cu^2+^, Fe^3+^, Sn^2+^, Pd^2+^, Ni^2+^, Ba^2+^, V^2+^, Ca^2+^, Co^2+^, Pt^4+^, Cd^2+^, Pb^2+^, Sr^2+^, Zn^2+^, and Hg^2+^ (25.0 μM for each); and similar species, such as (**C**) CF, KAN, PenG, STR, and DL−PEN (25.0 μM for each) on the relative fluorescence intensity of the biomass−based C−dots at 520 nm. (**D**) Fluorescence images of the biomass−based C−dots following incubation with the indicated substances (50.0 μM for each) under a 365 UV lamp. The concentration of the biomass-based C−dots was 25.0 μg/mL for TC and CTC and 10.0 μg/mL for OTC.

The selectivity of the proposed biomass−based C−dots toward TC and their analogues was also examined. Under the same conditions, the we measured the fluorescence response of the proposed biomass−based C−dots toward TCs (TC, CTC, and OTC), including common ions (e.g., Hg^2+^, Al^3+^, Ba^2+^, Ca^2+^, Cd^2+^, Co^2+^,Cu^2+^, Fe^3+^, Mg^2+^, Mn^2+^, Ni^2+^, Zn^2+^, V^3+^, V^2+^, Sr^2+^, Sn^2+^, Pt^4+^, Pd^2+^, Pb^2+^, Ag^+^, Cl^−^, I^−^, NO_3_^−^, PO_4_^3−^, and SO_4_^2−^), amino acids (L−ASP, Gly, L−Hcy, L−Phe, L−Lys, L−Cys, L−Val, L−Pro, and L−Tyr), GSH, CA, AA, and other antibiotics (e.g., CF, KAN, PenG, STR, and DL−pen), by comparing the fluorescence intensity of the biomass−based C−dots at 520 nm after incubation with these species over the course of 10.0 min. The corresponding results showed that only TC, CTC, and OTC could cause the remarkable fluorescence quenching of the biomass-based C−dots at 520 nm, whereas other species exhibited a negligible effect on the fluorescence intensity of the biomass−based C−dots (Figure 5). Additionally, in the presence of 50.0 μM TCs, 250.0 μM of these potentially interfering species did not affect the fluorescence response of the biomass-based C−dots toward TCs due to the unchanged fluorescence emission (Figures S8–S10). These findings clearly demonstrate that the biomass−based C−dots had an acceptable selectivity for TCs against the interfering species and could be suitable for application to environmental pollution traceability analysis of residual TCs in real samples.

### 3.4. Response Mechanism of Biomass-Based C−Dots toward TC Residues

**Figure 6:**
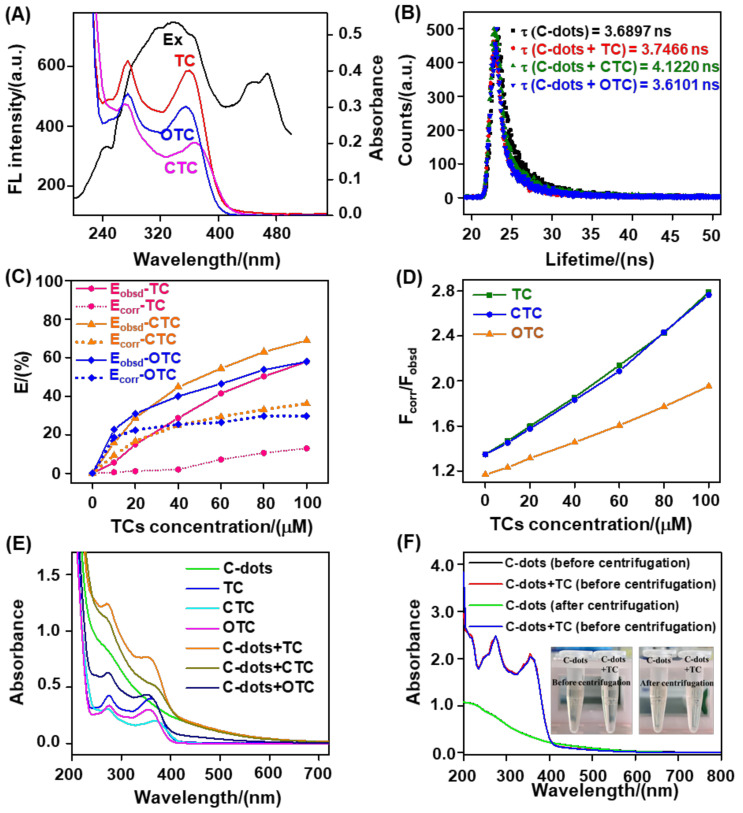
Profiling the fluorescent response mechanism of the biomass−based C−dots toward TCs in BR buffer (pH = 5.0 for TC and pH = 4.0 for CTC and OTC). (**A**) Fluorescence excitation (Ex) spectrum of C−dots and the absorption spectra of TC, OTC, and CTC. (**B**) Fluorescence lifetime decay curves of the biomass-based C−dots in the absence and presence of TC, OTC, and CTC. (**C**) Curves of *F*corr/*F*obsd versus TC concentration. *F*corr and *F*obsd correspond to the corrected and observed fluorescence intensities of the C−dots−TC reaction system at 520 nm, respectively. (**D**) Observed and corrected fluorescence decrease efficiency (E) of the biomass-based C−dots caused by TC, CTC, and OTC. *E* = (*F_0_* − *F*)/*F_0_*, *F_0_*, and *F* are assigned to the fluorescence intensities of the biomass-based C−dots at 520 nm in the absence and presence of TCs. (**E**) Absorption spectra of the biomass-based C−dots, TC, CTC, OTC, C−dots + TC, C−dots + CTC, and C−dots + OTC. (**F**) Absorption spectra of the biomass−based C−dots and C−dots + TC before and after centrifugation. Inset shows corresponding photographs as indicated by the absorption spectra. The concentrations of the biomass−based C−dots and TCs were 25.0 (for TC and CTC) or 10.0 (for OTC) μg/mL and 50.0 μM, respectively.

In general, the effect of optical overlapping between the fluorophore and quencher implied the occurrence of an inner filter effect (IFE) or fluorescence resonance energy transfer (FRET) process (Figure 6A) [47]. As an effective technique to determine the FRET process, the negligible fluorescence lifetime change of the biomass-based C−dots before and after incubation with TCs completely excluded the FRET role in TC−triggered C−dots fluorescence quenching (Figure 6B) [48]. To verify the IFE process between the biomass−based C−dots and TCs, the fluorescence decreased in the biomass−based C−dots was subsequently corrected by employing cuvette geometry, together with the absorption of biomass−based C−dots following incubation with TCs. The corresponding results showed that the observed decrease in efficiency (*E*obsd) was obviously higher than that of the corrected decrease efficiency (*E*cor, after removing the IFE), suggesting that IFE played a significant role in the TC−triggered decrease in the fluorescence of the biomass-based C−dots (Figure 6C,D, Figures S11 and S12, & Table S2) [22]. We also observed that no new absorption peak appeared in the TCs before and after incubation with the biomass-based C−dots (Figure 6E). Taking TC as an example, there was no precipitation in the C−dots + TC system before or after centrifugation (Figure 6F), indicating that no non−fluorescent ground−state complex was formed between C−dots and TCs. Considering these findings [49], IFE was the dominant fluorescence response mechanism of the biomass−based C−dots toward TCs.

### 3.5. Analysis of TCs in Real Samples

To examine the feasibility of the proposed biomass−based, C−dots-based analytical platform for monitoring of TCs in actual samples, its fluorescence response to TCs was evaluated in lake and tap water samples with the distinguished standard addition method. As shown in Table 1, relative standard deviations (R.S.D.s) from 0.79% to 6.12% implied a satisfactory accuracy of the proposed biomass−based C−dots platform in response to TCs in actual samples. Moreover, satisfactory recoveries in the range of 88.95%–112.07% illustrate the prominent repetitiveness of these biomass−based C−dots in response to TCs in real samples. According to the above analyses, the proposed biomass−based C−dots-based analytical platform is suitable for monitoring of residual TCs in complicated real samples with high reliability and accuracy, making it a promising prospect for environmental pollution traceability analysis.

## 4. Conclusions

In summary, we proposed a green, eco−friendly, high−efficiency, and economical strategy to transmute waste tobacco stems into valuable biomass−based fluorescence C−dots for application in environmental pollution traceability analysis of TCs residues. Through a simple one−pot hydrothermal method, C−dots derived from waste tobacco stems were successfully synthesized with the assistance of carbon black, presenting with a yellow−green fluorescence emission, a considerable solubility, and prominent stability against ionic strength and light irradiation. Moreover, the as−synthesized biomass−based C−dots exhibited a pH stimuli−responsive feature, as well as potential for the design of versatile pH-triggered response platforms. Furthermore, the as-synthesized biomass-based C−dots displayed a TC−concentration-dependent yellow−green fluorescence decreased via an IFE mechanism, enabling a label−free, highly sensitive and selective fluorescence assay toward TCs in real samples (i.e., tap and lake water). The LODs were calculated to be 1.328 nM for TC, 3.234 nM for CTC, and 9.881 nM for OTC, which are comparable with the LODs reported for TC residues by other assays. Overall, this study offers a new and sustainable direction for the highly valuable utilization of waste tobacco stems in the development of promising smart and green analytical platforms for environmental pollution traceability analysis.

## Figures and Tables

**Table 1:** Analysis of TCs with the proposed biomass−based C−dots−based analytical platform in real lake and tap water samples (n = 5).

Samples	TCs	Spiked (μM)	Found (μM)	Recovery (%, n = 5)	R.S.D. (%, n = 5)
Tap water		20.00	19.37	96.86	5.67
	TC	60.00	60.23	100.39	2.60
		80.00	78.61	98.27	1.56
		20.00	21.23	106.16	4.58
	CTC	60.00	61.84	103.58	4.22
		80.00	81.60	101.99	1.81
		60.00	60.51	100.86	4.04
	OTC	120.00	117.71	98.09	0.91
		180.00	179.52	99.73	0.79
Lake water		10.00	9.68	96.89	6.12
	TC	20.00	17.79	88.95	3.02
		30.00	31.95	106.48	4.66
		10.00	9.12	91.22	1.47
	CTC	20.00	20.33	101.64	3.18
		30.00	33.62	112.07	3.43
		20.00	18.97	94.86	4.40
	OTC	30.00	32.57	108.58	4.73
		50.00	53.11	106.22	1.57

## Data Availability

The data are available from the corresponding author upon reasonable request.

## Supplementary Materials

The following supporting information can be downloaded at: https://www.mdpi.com/article/10.3390/nano12183241/s1, Figure S1: FT−IR spectrum of the as−synthetized biomass−based C−dots; Figure S2: Zeta potential curve of the as−synthetized biomass−based C−dots in D.I. water; Figure S3: Concentration−dependent fluorescence emission spectra of the as−synthetized biomass−based C−dots in D.I. water.; Figure S4: Fluorescence emission spectra of the as−synthetized biomass−based C−dots at the same concentration in different solvents, such as H_2_O, DMSO, DMF, THF, CH_3_COCH_3_, CH_3_CH_2_OH, and CH_3_CN.; Figure S5: Conditional optimization experiment for TC detection; Figure S6: Conditional optimization experiment for CTC detection; Figure S7: Conditional optimization experiment for OTC detection; Figure S8: Influence of various interferences on TC detection; Figure S9: Influence of various interferences on CTC detection; Figure S10: Influence of various interferences on OTC detection; Figure S11: IFE action during fluorescence decrease of the biomass−based C−dots at 520 nm; Figure S12: IFE-corrected UV-vis absorption of varying concentrations of TCs; Table S1: Comparison of the proposed biomass−based, C−dots−based method with other reported fluorescence assays for the detection of TCs; Table S2: Influence of IFE behaviors of TCs on the fluorescence decrease in the biomass−based C−dots at 520 nm. [50,51,52,53,54,55,56] cited in the Supplementary Materials.

## References

[1] Baragau I.-A., Power N.P., Morgan D.J., Lobo R.A., Roberts C.S., Titirici M.-M., Middelkoop V., Diaz Z., Kellici S. (2021). Efficient Continuous Hydrothermal Flow Synthesis of Carbon Quantum Dots from a Targeted Biomass Precursor for on-off Metal Ions Nanosensing. ACS Sustain. Chem. Eng..

[2] Ramanan V., Siddaiah B., Raji K., Ramamurthy P. (2018). Green Synthesis of Multifunctionalized, Nitrogen-doped, Highly Fluorescent Carbon Dots from Waste Expanded Polystyrene and its Application in the Fluorimetric Detection of Au^3+^ Ions in Aqueous Media. ACS Sustain. Chem. Eng..

[3] Samanta S.K., Dey N., Kumari N., Biswakarma D., Bhattacharya S. (2019). Multimodal Ion Sensing by Structurally Simple Pyridine-end Oligo p-phenylenevinylenes for Sustainable Detection of Toxic Industrial Waste. ACS Sustain. Chem. Eng..

[4] Shao T., Liu L., Li Y., Zhang X., Deng Z., Huo L., Gao S. (2021). Highly Sensitive and Selective Detection of Long-chain Alcohol Vapors based on Keel-type ZnO Fibers Derived from Waste Cigarette Butts. ACS Sustain. Chem. Eng..

[5] Zhao J., Huang M., Zhang L., Zou M., Chen D., Huang Y., Zhao S. (2017). Unique Approach to Develop Carbon Dot-based Nanohybrid Near-infrared Ratiometric Fluorescent Sensor for the Detection of Mercury Ions. Anal. Chem..

[6] Zairov R.R., Dovzhenko A.P., Sarkanich K.A., Nizameev I.R., Luzhetskiy A.V., Sudakova S.N., Podyachev S.N., Burilov V.A., Vatsouro I.M., Vomiero A. (2021). Single Excited Dual Band Luminescent Hybrid Carbon Dots-Terbium Chelate Nanothermometer. Nanomaterials.

[7] Kamyshnikov A.G., Zaripov A.T., Beregovoy A.N., Ibatullin R.R., Zairov R.R., Dovzhenko A.P. (2021). Carbon quantum dots used as tracers in ecological, hydrogeological monitoring and reservoir management. Neftyanoe Khozyaystvo Oil Ind..

[8] Huang X., Liu J., Zhao B., Bai Y., Peng Z., Zhou J., Wang C., Zhao X., Han S., Zhang C. (2022). One-step Synthesis of Biomass-based Carbon Dots for Detection of Metal Ions and Cell Imaging. Front. Energy Res..

[9] Liang C., Xie X., Zhang D., Feng J., Lu S., Shi Q. (2021). Biomass Carbon Dots Derived from Wedelia Trilobata for the Direct Detection of Glutathione and Their Imaging Application in Living Cells. J. Mater. Chem. B.

[10] Wareing T.C., Gentile P., Phan A.N. (2021). Biomass-based Carbon Dots: Current Development and Future Perspectives. ACS Nano.

[11] Chellasamy G., Arumugasamy S.K., Govindaraju S., Yun K. (2022). Green Synthesized Carbon Quantum Dots from Maple Tree Leaves for Biosensing of Cesium and Electrocatalytic Oxidation of Glycerol. Chemosphere.

[12] Yadav P.K., Singh V.K., Chandra S., Bano D., Kumar V., Talat M., Hasan S.H. (2019). Green Synthesis of Fluorescent Carbon Quantum Dots from Azadirachta Indica Leaves and Their Peroxidase-mimetic Activity for the Detection of H_2_O_2_ and Ascorbic Acid in Common Fresh Fruits. ACS Biomater. Sci. Eng..

[13] Li Z., Wang Q., Zhou Z., Zhao S., Zhong S., Xu L., Gao Y., Cui X. (2021). Green Synthesis of Carbon Quantum Dots from Corn Stalk Shell by Hydrothermal Approach in Near-critical Water and Applications in Detecting and Bioimaging. Microchem. J..

[14] Wang Y., He Q., Zhao X., Yuan J., Zhao H., Wang G., Li M. (2022). Synthesis of Corn Straw-based Graphene Quantum Dots (GQDs) and Their Application in PO43- Detection. J. Environ. Chem. Eng..

[15] Xue M., Zhan Z., Zou M., Zhang L., Zhao S. (2016). Green Synthesis of Stable and Biocompatible Fluorescent Carbon Dots from Peanut Shells for Multicolor Living Cell Imaging. New J. Chem..

[16] Yao L., Zhao M.-M., Luo Q.-W., Zhang Y.-C., Liu T.-T., Yang Z., Zeng K.-W. (2022). Carbon Quantum Dots-based Nanozyme from Coffee Induces Cancer Cell Ferroptosis to Activate Antitumor Immunity. ACS Nano.

[17] Chen D., Zhao J., Zhang L., Liu R., Huang Y., Lan C., Zhao S. (2018). Capsicum-derived Biomass Quantum Dots Coupled with Alizarin Red S as An Inner-filter-mediated Illuminant Nanosystem for Imaging of Intracellular Calcium Ions. Anal. Chem..

[18] Zhou J., Sheng Z., Han H., Zou M., Li C. (2012). Facile Synthesis of Fluorescent Carbon Dots Using Watermelon Peel as A Carbon Source. Mater. Lett..

[19] Tuzzin G., Godinho M., Dettmer A., Zattera A.J. (2016). Nanofibrillated Cellulose from Tobacco Industry Wastes. Carbohydr. Polym..

[20] Chen H., Zhu C., Chen F., Xu J., Jiang X., Wu Z., Ding X., Fan G.-C., Shen Y., Ye Y. (2020). Profiling the Interaction of Al (III)-GFLX Complex, A Potential Pollution Risk, with Bovine Serum Albumin. Food Chem. Toxicol..

[21] Shen Y., Wei Y., Zhu C., Cao J., Han D.-M. (2022). Ratiometric Fluorescent Signals-driven Smartphone-based Portable Sensors for Onsite Visual Detection of Food Contaminants. Coord. Chem. Rev..

[22] Ye Y., Wu T., Jiang X., Cao J., Ling X., Mei Q., Chen H., Han D., Xu J.-J., Shen Y. (2020). Portable Smartphone-based QDs for the Visual Onsite Monitoring of Fluoroquinolone Antibiotics in Actual Food and Environmental Samples. ACS Appl. Mater. Interfaces.

[23] Shen Y., Wei Y., Chen H., Wu Z., Ye Y., Han D.-M. (2022). Liposome-encapsulated Aggregation-induced Emission Fluorogen Assisted with Portable Smartphone for Dynamically on-site Imaging of Residual Tetracycline. Sens. Actuators B Chem..

[24] Shen Y., Wei Y., Liu Z., Nie C., Ye Y. (2022). Engineering of 2D Artificial Nanozyme-based Blocking Effect-triggered Colorimetric Sensor for Onsite Visual Assay of Residual Tetracycline in Milk. Microchim. Acta.

[25] Moga A., Vergara-Barberán M., Lerma-García M.J., Carrasco-Correa E.J., Herrero-Martínez J.M., Simó-Alfonso E.F. (2021). Determination of Antibiotics in Meat Samples using Analytical Methodologies: A review. Compr. Rev. Food Sci. Food Saf..

[26] Liu X., Huang D., Lai C., Zeng G., Qin L., Zhang C., Yi H., Li B., Deng R., Liu S. (2018). Recent Advances in Sensors for Tetracycline Antibiotics and Their Applications. Trends Anal. Chem..

[27] Zong L.-P., Li J., Shu G., Liu X., Marks R.S., Zhang X.-J., Cosnier S., Shan D. (2021). Rational Design of a Highly Dispersed Fe-N-C Nanosheet with 1,10-Phenanthroline-2,9-Dicarboxylic Acid as A Preorganized Ligand: Boosted Electrochemiluminescence Detection of Tetracycline. Anal. Chem..

[28] Wang X., Li L., Jiang H., Zhangsun H., Wang Q., Sun X., Wang L. (2022). Highly Selective and Sensitive Fluorescence Detection of Tetracyclines based on Novel Tungsten Oxide Quantum Dots. Food Chem..

[29] Li H., Wu J., Meng F., Li A. (2021). Immunochromatographic Assay for the Detection of Antibiotics in Animal-derived Foods: A review. Food Control.

[30] Shamsutdinova N., Zairov R., Mustafina A., Podyachev S., Sudakova S., Nizameev I., Kadirov M., Amirov R. (2015). Interfacial interactions of hard polyelectrolyte-stabilized luminescent colloids with substrates. Colloids Surf. A Physicochem. Eng. Asp..

[31] Ehrat F., Bhattacharyya S., Schneider J., Löf A., Wyrwich R., Rogach A.L., Stolarczy J.K., Urban A.S., Feldmann J. (2017). Tracking the Source of Carbon Dot Photoluminescence: Aromatic Domains versus Molecular Fluorophores. Nano Lett..

[32] Mao Y.-N., Wang J., Gao Y.-H., Zhao T.-T., Xu S.-H., Luo X.-L. (2021). Progress in Synthesis and Sensing Imaging of Biomass-based Carbon Quantum dots. Chinese J. Anal. Chem..

[33] Wang G., Guo Q., Chen D., Liu Z., Zheng X., Xu A., Yang S., Ding G. (2018). Facile and Highly Effective Synthesis of Controllable Lattice Sulfur-doped Graphene Quantum Dots via Hydrothermal Treatment of Durian. ACS Appl. Mater. Interfaces.

[34] Zhang X., Huang H., Liu J., Liu Y., Kang Z. (2013). Carbon Quantum Dots Serving as Spectral Converters Through Broadband Upconversion of Near-infrared Photons for Photoelectrochemical Hydrogen Generation. J. Mater. Chem. A.

[35] Feng J., Wang W., Hai X., Yu Y.L., Wang J.H. (2016). Green preparation of nitrogen doped carbon dots derived from silkworm chrysalis for cell imaging. J. Mater. Chem. B.

[36] Liu R., Wu D., Feng X., Müllen K. (2011). Bottom-up fabrication of photoluminescent graphene quantum dots with uniform morphology. J. Am. Chem. Soc..

[37] Shen Y., Wu T., Zhang Y., Ling N., Zheng L., Zhang S.-L., Xu J.-J., Ye Y. (2020). Engineering of A Dual-recognition Ratiometric Fluorescent Nanosensor with A Remarkably Large stokes Shift for Accurate Tracking of Pathogenic Bacteria at the Single-cell Level. Anal. Chem..

[38] Hu Y., Gao Z. (2020). Yellow Emissive Se,N-codoped Carbon Dots toward Sensitive Fluorescence Assay of Crystal Violet. J. Hazard. Mater..

[39] Miao H., Wang Y., Yang X. (2018). Carbon dots derived from Tobacco for Visually Distinguishing and Detecting Three Kinds of Tetracyclines. Nanoscale.

[40] Sun X., Wang C., Li P., Shao Z., Xia J., Liu Q., Shen F., Fang Y. (2022). The Facile Synthesis of Nitrogen and Sulfur Co-doped Carbon Dots for Developing a Powerful “On-off-on” Fluorescence Probe to Detect Glutathione in Vegetables. Food Chem..

[41] Shi W., Guo F., Han M., Yuan S., Guan W., Li H., Huang H., Liu. Y., Kang Z. (2017). N,S co-doped Carbon Dots as A Stable Bio-imaging Probe for Detection of Intracellular Temperature and Tetracycline. J. Mater. Chem. B.

[42] Yang Y., Wei Q., Zou T., Kong Y., Su L., Ma D., Wang Y. (2020). Dual-emission Ratiometric Fluorescent Detection of Dinotefuran based on Sulfur-doped Carbon Quantum Dots and Copper Nanocluster Hybrid. Sens. Actuators B Chem..

[43] Gan Z., Xu H., Hao Y. (2016). Mechanism for excitation-dependent photoluminescence from graphene quantum dots and other graphene oxide derivates: Consensus, debates and challenges. Nanoscale.

[44] Long Y., Zhou C., Zhang Z. (2012). Shifting and non-shifting fluorescence emitted by carbon nanodots. J. Mater. Chem..

[45] Guo Y., Wang Z., Shao H., Jiang X. (2013). Hydrothermal Synthesis of Highly Fluorescent Carbon Nanoparticles from Sodium Citrate and Their Use for the Detection of Mercury Ions. Carbon.

[46] Ti M., Li Y., Li Z., Zhao D., Wu L., Yuan L., He Y. (2021). A Ratiometric Nanoprobe based on Carboxylated Graphitic Carbon Nitride Nanosheets and Eu3+ for the Detection of Tetracyclines. Analyst.

[47] Zhai W., Wang C., Yu P., Wang Y., Mao L. (2014). Single-layer MnO_2_ Nanosheets Suppressed Fluorescence of 7-Hydroxycoumarin: Mechanistic Study and Application for Sensitive Sensing of Ascorbic Acid in Vivo. Anal. Chem..

[48] Shen Y., Sun Y., Yan R., Chen E., Wang H., Ye D., Xu J.-J., Chen H.-Y. (2017). Rational Engineering of Semiconductor QDs Enabling Remarkable O-1(2) Production for Tumor-targeted Photodynamic Therapy. Biomaterials.

[49] Shen Y., Liu S., Wang J., Li D., Wang X., He Y. (2013). A Sensitive Assay of Chelerythrine Using a Fluorescence Quenching Approach with Glutathione Capped CdTe/CdS Quantum Dots as A Probe. Anal. Methods.

[50] Magde D., Wong R., Seybold P.G. (2002). Fluorescence quantum yields and their relation to lifetimes of rhodamine 6G and fluorescein in nine solvents: Improved absolute standards for quantum yields. Photochem. Photobiol..

[51] Fan Y.J., Su M., Shi Y.E., Liu X.T., Shen S.G., Dong J.X. (2022). A ratiometric fluorescent sensor for tetracyclines detection in meat based on pH-dependence of targets with lanthanum-doped carbon dots as probes. Anal. Bioanal.Chem..

[52] Li C., Yang W., Zhang Y., Tang W., Yue T., Li Z. (2020). A 3D hierarchical dual-metal–organic framework heterostructure up-regulating the pre-concentration effect for ultrasensitive fluorescence detection of tetracycline antibiotics. J. Mater. Chem. C.

[53] Yang K., Jia P., Hou J., Bu T., Sun X., Liu Y., Wang L. (2020). Innovative dual-emitting ratiometric fluorescence sensor for tetracyclines detection based on boron nitride quantum dots and europium ions. ACS Sustain. Chem. Eng..

[54] Hu X., Zhao Y., Dong J., Liu C., Qi Y., Fang G., Wang S. (2021). A strong blue fluorescent nanoprobe based on Mg/N co-doped carbon dots coupled with molecularly imprinted polymer for ultrasensitive and highly selective detection of tetracycline in animal-derived foods. Sens. Actuators B Chem..

[55] Li C., Zeng C., Chen Z., Jiang Y., Yao H., Yang Y., Wong W.T. (2020). Luminescent lanthanide metal-organic framework test strip for immediate detection of tetracycline antibiotics in water. J. Hazard. Mater..

[56] Li R., Wang W., El-Sayed E.S.M., Su K., He P., Yuan D. (2021). Ratiometric fluorescence detection of tetracycline antibiotic based on a polynuclear lanthanide metal–organic framework. Sens. Actuators B Chemical.

