# Supplementary material for: High-Efficiency Utilization of Waste Tobacco Stems to Synthesize Novel Biomass-Based Carbon Dots for Precise Detection of Tetracycline Antibiotic Residues
Source: Nanomaterials (Basel). 2022 Sep 18;12(18):3241. doi: 10.3390/nano12183241 (PMC9503805; doi:10.3390/nano12183241)
Supplement: Supplementary file 1 [file nanomaterials-12-03241-s001.zip › nanomaterials-1900357-supplementary.pdf]

nanomaterials

Supplement Materials

High-Efficiency Utilization of Waste Tobacco Stems to Synthesize Novel Biomass-Based Carbon Dots for Precise Detection of Tetracycline Antibiotic Residues

Hui Yang [1,2,3], Yunlong Wei [4], Xiufang Yan [5], Chao Nie [4], Zhenchun Sun [2], Likai Hao [1,*] and Xiankun Su [2,*]

[1] State Key Laboratory of Environmental Geochemistry, Institute of Geochemistry, Chinese Academy of Sciences, Guiyang 550081, China
[2] Guizhou Academy of Tobacco Science, Guiyang 550081, China
[3] University of Chinese Academy of Sciences, Beijing 100049, China
[4] School of Food & Biological Engineering, Key Laboratory for Agricultural Products Processing of Anhui Province, Hefei University of Technology, Hefei 230009, China
[5] Key Laboratory of Tobacco Quality Research of Guizhou Province, College of Tobacco Science, Guizhou University, Guiyang 550025, China
* Correspondence: haolikai@mail.gyig.ac.cn (L.H.); xiankunsu@163.com(X.S.)

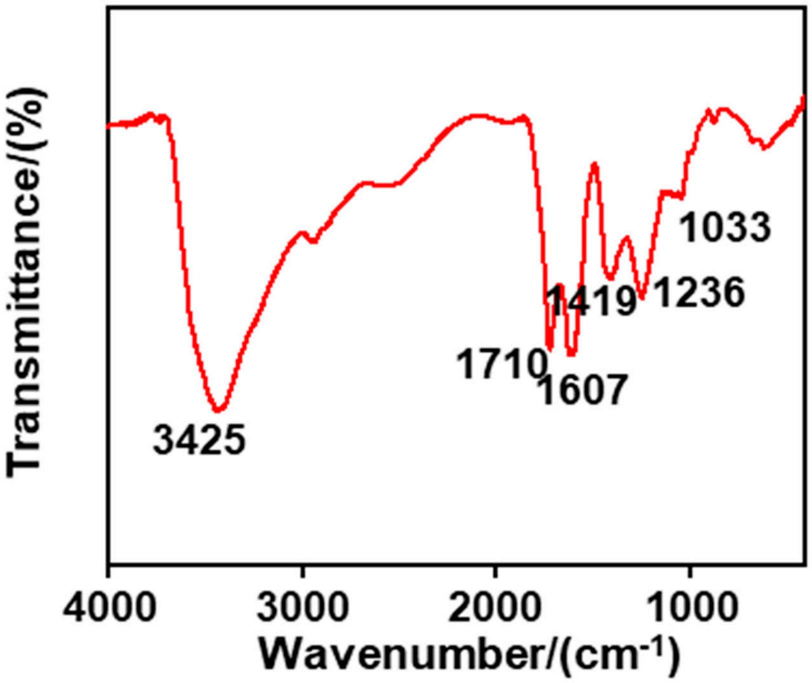

Figure S1. FT–IR spectrum of this as–synthetized biomass–based C–dots.

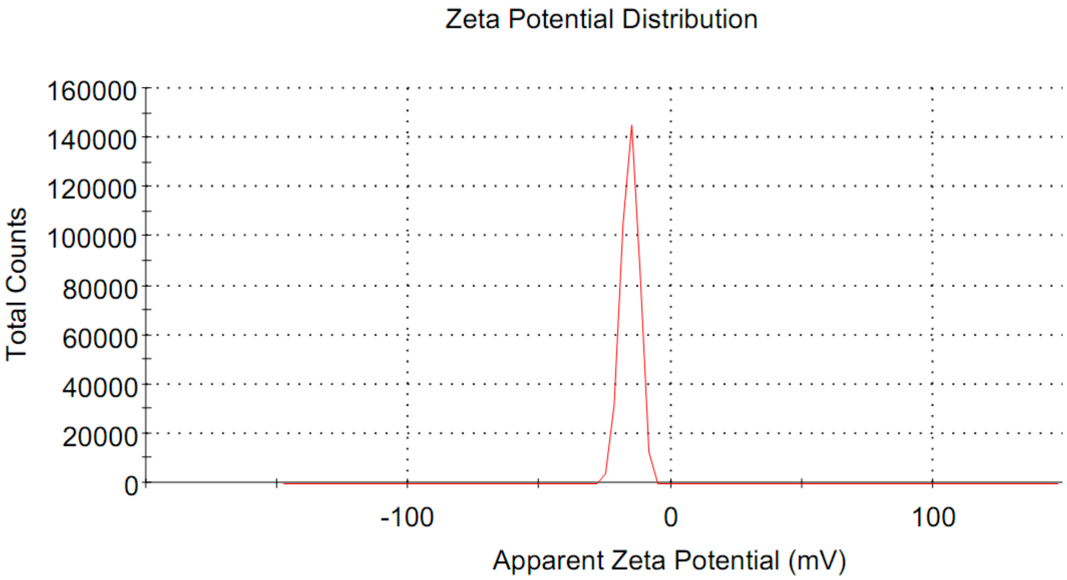

Figure S2. Zeta potential curve of this as−synthetized biomass−based C−dots in D.I. water.

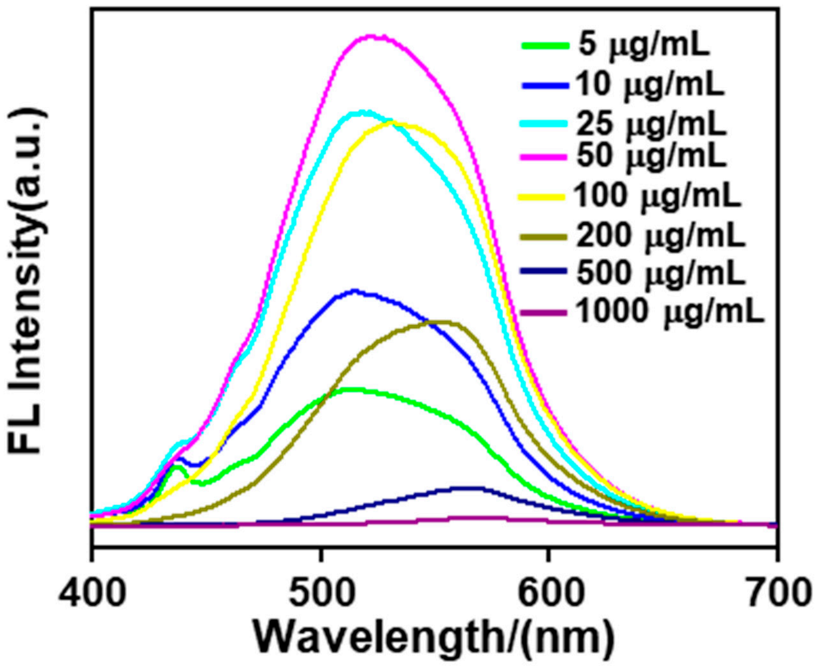

Figure S3. The concentration−dependent fluorescence emission spectra of this as-synthetized biomass−based C−dots in D.I. water.

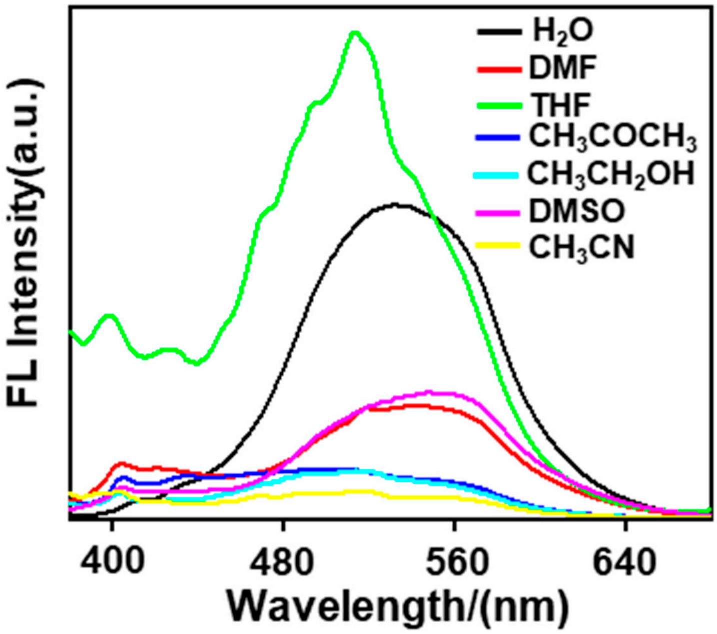

Figure S4. Fluorescence emission spectra of this as-synthetized biomass-based C-dots at the same concentration in different solvents, such as H_2O, DMSO, DMF, THF, CH_3COCH_3, CH_3CH_2OH, and CH_3CN.

Note S1. The fluorescence quantum yield (Y_a) of this as–synthetized biomass–based C–dots was calculated by the following formula [1]:

$$Y_{\mathrm{a}} = Y_b \times \left(\frac{F_a}{F_b}\right) \times \left(\frac{A_b}{A_a}\right) \times \left(\frac{n_a^2}{n_b^2}\right)$$

In this expression, Y_a and Y_b represented the fluorescence quantum yields of this as–synthetized biomass-based C-dots in TFH and rhodamine 6G in CH_3CH_2OH, respectively. F_a and F_b were the fluorescence intensity of this as–synthetized biomass-based C–dots in THF and rhodamine 6G in CH_3CH_2OH, respectively. A_a and A_b were the absorbance of this as–synthetized biomass–based C–dots in TFH and rhodamine 6G in CH_3CH_2OH, respectively. n_a and n_b were the refractive index of this as-synthetized biomass-based C–dots in TFH (n_a = 1.4050) and rhodamine 6G in CH_3CH_2OH (n_b = 1.3290), respectively. The fluorescence quantum yield of rhodamine 6G was 95.0% in CH_3CH_2OH. The fluorescence quantum yield of this as-synthetized biomass-based C-dots could be calculated as 19.45% in THF.

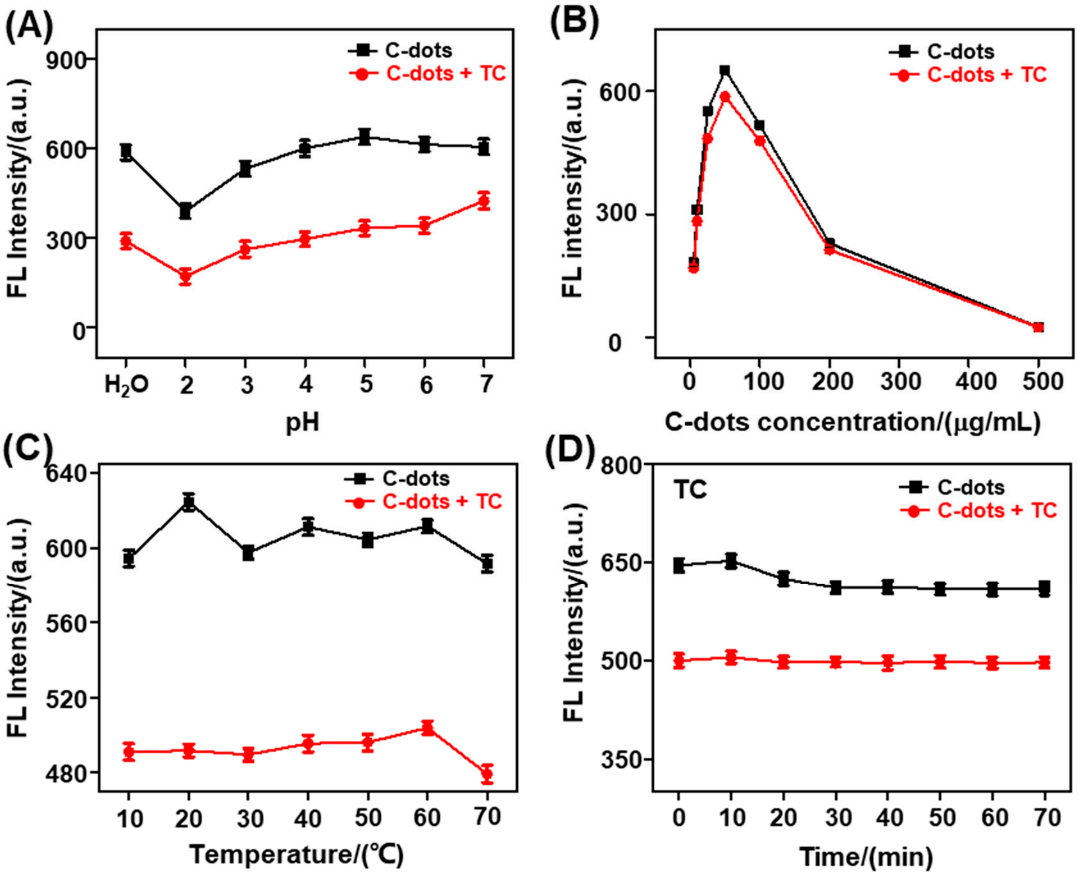

Figure S5. Conditional optimization experiment for TC detection. (**A**) Effect of different pH on the fluorescence burst of TC on the prepared C−dots. Reaction conditions: 1.0 mL C−dots (50.0 μg/mL) and 1.0 mL TC (50.0 μM) were incubated in BR buffer (pH: 2.0–7.0) at 20.0 °C for 10.0 min. (**B**) Effect of varying C−dots concentration on the fluorescence quenching of the as−prepared C−dots by TC. Reaction conditions: 1.0 mL C−dots and 1.0 mL TC (50.0 μM) were incubated in BR buffer (pH = 5.0) at 20.0 °C for 10.0 min. (**C**) Effect of varying temperature on the fluorescence burst of TC on the prepared C−dots. Reaction conditions: 1.0 mL C−dots (50.0 μg/mL) and 1.0 mL TC (50.0 μM) were incubated in BR buffer (pH = 5.0) for 10.0 min. (**D**) Effect of varying reaction time on the fluorescence burst of TC on the prepared C−dots. Reaction conditions: 1.0 mL C−dots (50.0 μg/mL) and 1.0 mL TC (50.0 μM) were incubated in BR buffer (pH = 5.0) at 20.0 °C.

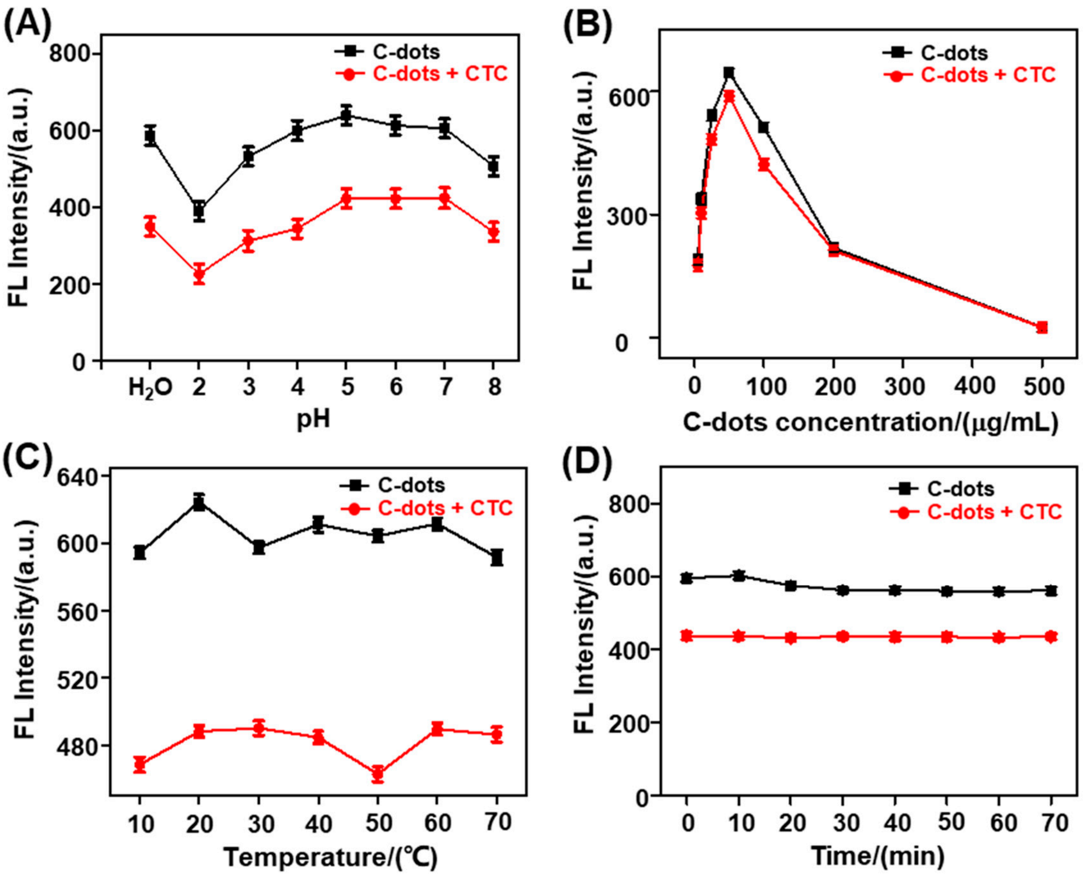

Figure S6. Conditional optimization experiment for CTC detection. (**A**) Effect of different pH on the fluorescence burst of CTC on the prepared C–dots. Reaction conditions: 1.0 mL C–dots (50.0 μg/mL) and 1.0 mL CTC (50.0 μM) were incubated in BR buffer (pH: 2.0–8.0) at 50.0 °C for 10.0 min. (**B**) Effect of varying C–dots concentration on the fluorescence quenching of the as-prepared C–dots by CTC. Reaction conditions: 1.0 mL C–dots and 1.0 mL CTC (50.0 μM) were incubated in BR buffer (pH = 4.0) at 50.0 °C for 10.0 min. (**C**) Effect of varying temperature on the fluorescence burst of CTC on the prepared C–dots. Reaction conditions: 1.0 mL C–dots (50 μg/mL) and 1.0 mL CTC (50.0 μM) were incubated in BR buffer (pH = 4.0) for 10.0 min. (**D**) Effect of varying reaction time on the fluorescence burst of CTC on the prepared C–dots. Reaction conditions: 1.0 mL C–dots (50.0 μg/mL) and 1.0 mL CTC (50.0 μM) were incubated in BR buffer (pH = 4.0) at 50.0 °C.

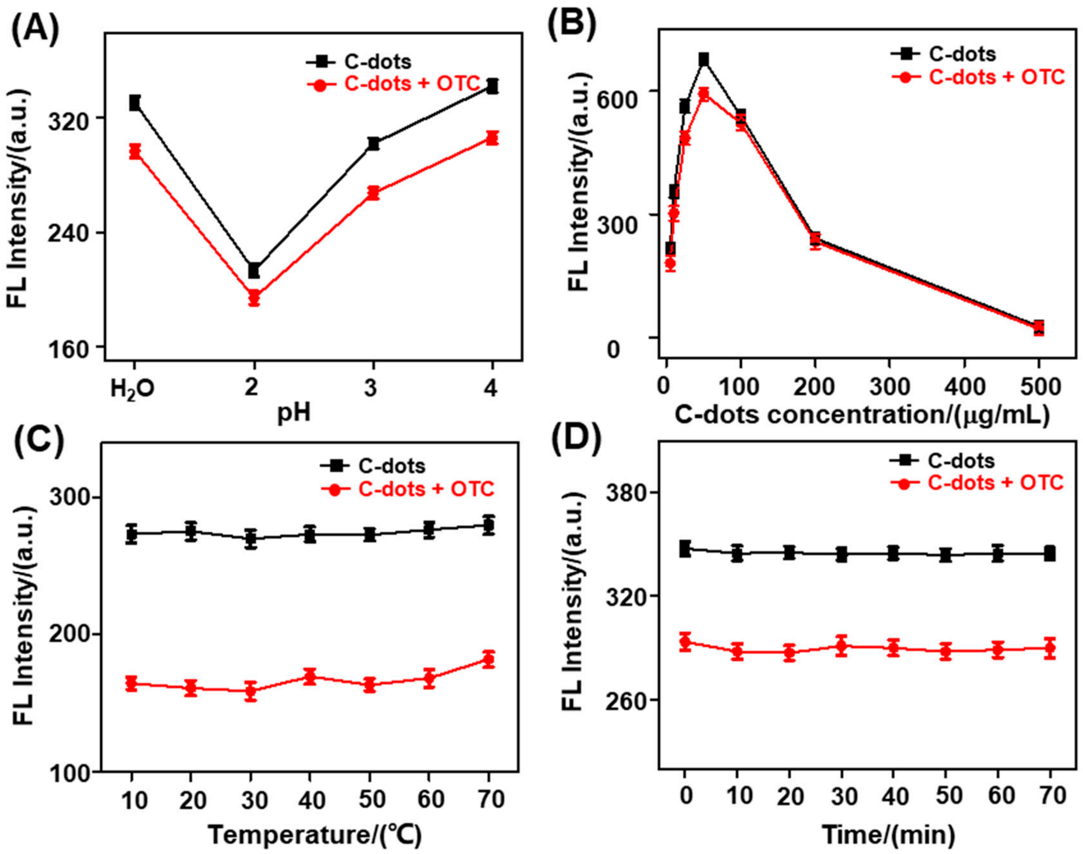

Figure S7. Conditional optimization experiment for OTC detection. (**A**) Effect of different pH on the fluorescence burst of OTC on the prepared C–dots. Reaction conditions: 1.0 mL C–dots (20.0 μg/mL) and 1.0 mL OTC (50.0 μM) were incubated in BR buffer (pH: 2.0 – 4.0) at 20.0 °C for 10.0 min. (**B**) Effect of varying C–dots concentration on the fluorescence quenching of the as-prepared C–dots by OTC. Reaction conditions: 1.0 mL C–dots and 1.0 mL OTC (50.0 μM) were incubated in BR buffer (pH = 4.0) at 20.0 °C for 10.0 min. (**C**) Effect of varying temperature on the fluorescence burst of OTC on the prepared C–dots. Reaction conditions: 1.0 mL C–dots (20.0 μg/mL) and 1.0 mL OTC (50.0 μM) were incubated in BR buffer (pH = 4.0) for 10.0 min. (**D**) Effect of varying reaction time on the fluorescence burst of OTC on the prepared C–dots. Reaction conditions: 1.0 mL C–dots (20.0 μg/mL) and 1.0 mL OTC (50.0 μM) were incubated in BR buffer (pH = 4.0) at 20.0 °C.

Table S1. Comparison of this proposed biomass-based C–dots-based method with other reported fluorescence assays for the detection of TCs.

Fluorescent materials	Linear range	Detection limit	Ref.
Eu^{3+}@AIEgen	0.0961–10.0 μM	28.83 nM (TC)	[2]
La–CDs	0–805.20 μM (OTC)<br>0–1039.50 μM (TC)	42.90 μM (OTC)<br>83.30 μM (TC)	[3]
Al–MOF@Mo/Zn–MOF	0–55 μM	0.58 nM (OTC)<br>0.86 nM (CTC)	[4]
C–dots	15.5–6000 nM (TC)<br>20–2000 nM (OTC)<br>20–200 nM (CTC)	5.18 nM (TC)<br>6.06 nM (OTC)<br>14 nM (CTC)	[5]
B–NQDs–Eu^{3+}	2.5–50 μM (TC)<br>0–50 μM (OTC)	0.019 μM (TC)<br>0.104 μM (OTC)	[6]
Mg,N–CDs@MIP	5–100 ng/mL	0.79 ng/mL (TC)	[7]
LnMOF	0–50 μM	2.77 nM (TC)<br>1.95 nM (OTC)	[8]
Ln–MOF	0.06–10 μg/mL (TC)<br>0.1–40 μg/mL (CTC)<br>0.1–20 μg/mL (OTC)	8 ng/mL (TC)<br>40 ng/mL (CTC)<br>20 ng/mL (OTC)	[9]

This biomass-based C–dots	0.004–100 μM (TC)<br>0.011–100 μM (CTC)<br>0.033–200 μM (OTC)	1.328 nM (TC)<br>3.234 nM (CTC)<br>9.881 nM (OTC)	This work

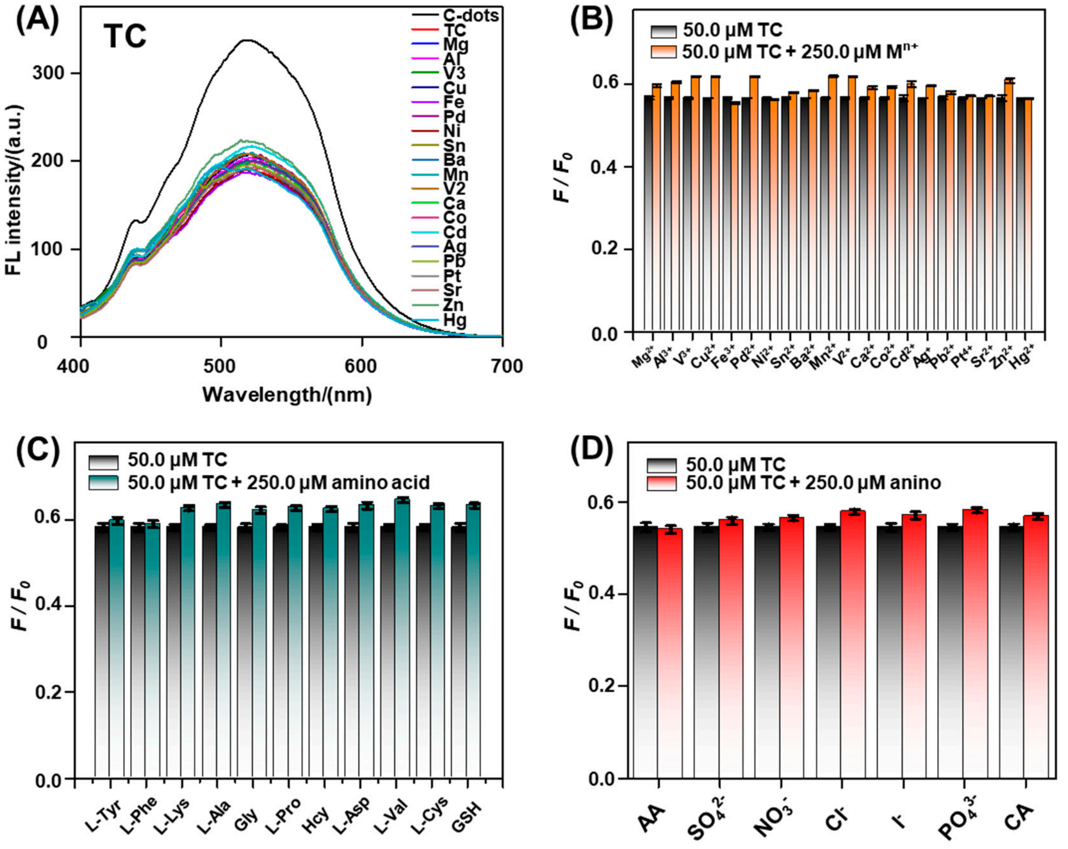

Figure S8. Influence by various interferences (**A**) fluorescence spectra variation of C–dots with TC (take metal ions as an example). Reaction conditions: 1.0 mL C–dots (50.0 μg/mL),0.5 mL TC (200.0 μM) and 0.5 mL M^{n+} (1000.0 μM) were incubated in BR buffer (pH = 5.0) at 20.0 °C for 10.0 min. Influence by various interferences on the fluorescence intensity (**B**) Metal ions (Mg^{2+}, Al^{3+}, Mn^{2+}, V^{3+}, Ag^{+}, Cu^{2+}, Fe^{3+}, Sn^{2+}, Pd^{2+}, Ni^{2+}, Ba^{2+}, V^{2+}, Ca^{2+}, Co^{2+}, Pt^{4+}, Cd^{2+}, Pb^{2+}, Sr^{2+}, Zn^{2+}, and Hg^{2+}). Reaction conditions: 1.0 mL C–dots (50.0 μg/mL), 0.5 mL TC (200.0 μM) and 0.5 mL M^{n+} (1000.0 μM) were incubated in BR buffer (pH = 5.0) at 20.0 °C for 10.0 min. (**C**) Common amino acids (L–Tyr, L–Phe, L–Lys, L–Ala, Gly, L–Pro, L–Hcy, L–Asp, L–Val, L–Cys, and GSH). Reaction conditions: 1.0 mL C–dots (50.0 μg/mL), 0.5 mL TC (200.0 μM) and 0.5 mL amino acids (1000.0 μM) were incubated in BR buffer (pH = 5.0) at 20.0 °C for 10.0 min. (**D**) Common anions and acids (AA, SO_4^{2-}, NO_3^-, Cl^-, I^-, PO_4^{3-}, and CA). Reaction conditions: 1.0 mL C–dots (50.0 μg/mL), 0.5 mL TC (200.0 μM) and 0.5 mL amino acid (1000.0 μM) were incubated in BR buffer (pH = 5.0) at 20.0 °C for 10.0 min.

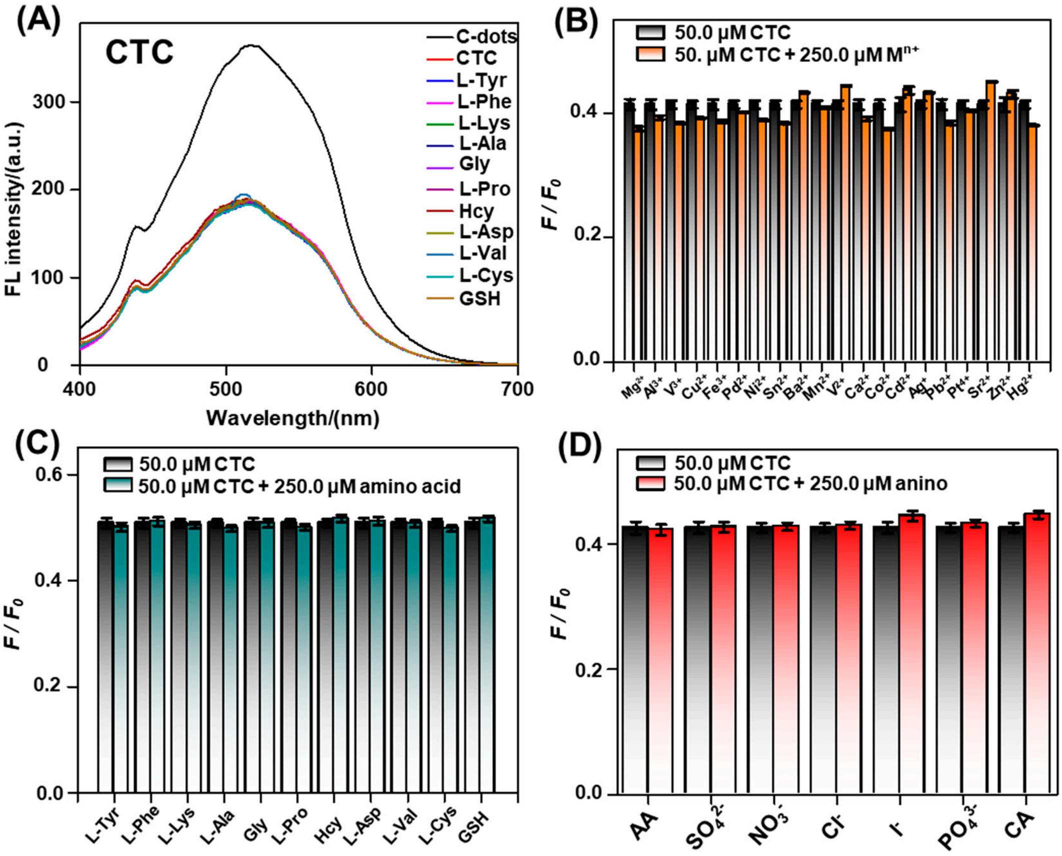

Figure S9. Influence by various interferences (**A**) fluorescence spectra variation of C−dots with CTC (take metal ions as an example). Reaction conditions: 1.0 mL C-dots (50.0 μg/mL), 0.5 mL CTC (200.0 μM) and 0.5 mL M^{n+} (1000.0 μM) were incubated in BR buffer (pH = 4.0) at 50.0 °C for 10.0 min. Influence by various interferences on the fluorescence intensity (**B**) Metal ions (Mg^{2+}, Al^{3+}, Mn^{2+}, V^{3+}, Ag^{+}, Cu^{2+}, Fe^{3+}, Sn^{2+}, Pd^{2+}, Ni^{2+}, Ba^{2+}, V^{2+}, Ca^{2+}, Co^{2+}, Pt^{4+}, Cd^{2+}, Pb^{2+}, Sr^{2+}, Zn^{2+}, and Hg^{2+}). Reaction conditions: 1.0 mL C−dots (50.0 μg/mL), 0.5 mL CTC (200.0 μM) and 0.5 mL M^{n+} (1000.0 μM) were incubated in BR buffer (pH = 4.0) at 50.0 °C for 10.0 min. (**C**) Common amino acids (L−Tyr, L−Phe, L−Lys, L−Ala, Gly, L-Pro, L−Hcy, L−Asp, L−Val, L−Cys, and GSH). Reaction conditions: 1.0 mL C−dots (50.0 μg/mL), 0.5 mL CTC (200.0 μM) and 0.5 mL amino acids (1000.0 μM) were incubated in BR buffer (pH = 4.0) at 50.0 °C for 10.0 min. (**D**) Common anions and acids (AA, SO_4^{2-}, NO_3^-, Cl^-, I^-, PO_4^{3-}, and CA). Reaction conditions: 1.0 mL C−dots (50.0 μg/mL), 0.5 mL CTC (200.0 μM) and 0.5 mL anino acid (1000.0 μM) were incubated in BR buffer (pH = 4.0) at 50.0 °C for 10.0 min.

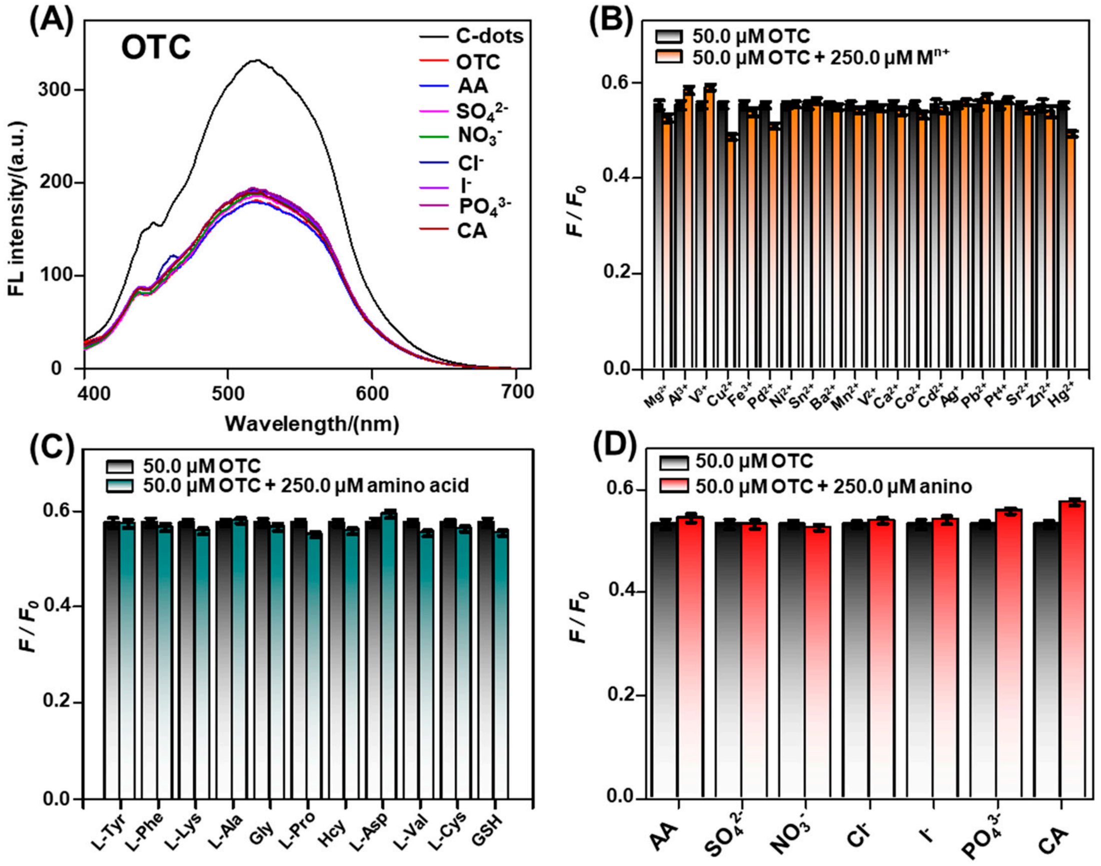

Figure S10. Influence by various interferences (**A**) fluorescence spectra variation of C−dots with OTC (take metal ions as an example). Reaction conditions: 1.0 mL C−dots (20.0 μg/mL), 0.5 mL OTC (200.0 μM) and 0.5 mL M^{n+} (1000.0 μM) were incubated in BR buffer (pH = 4.0) at 20.0 °C for 10.0 min. Influence by various interferences on the fluorescence intensity (**B**) Metal ions (Mg^{2+}, Al^{3+}, Mn^{2+}, V^{3+}, Ag^{+}, Cu^{2+}, Fe^{3+}, Sn^{2+}, Pd^{2+}, Ni^{2+}, Ba^{2+}, V^{2+}, Ca^{2+}, Co^{2+}, Pt^{4+}, Cd^{2+}, Pb^{2+}, Sr^{2+}, Zn^{2+}, and Hg^{2+}). Reaction conditions: 1.0 mL C−dots (20.0 μg/mL), 0.5 mL OTC (200.0 μM) and 0.5 mL M^{n+} (1000.0 μM) were incubated in BR buffer (pH = 4.0) at 20.0 °C for 10.0 min. (**C**) Common amino acids (L−Tyr, L−Phe, L−Lys, L−Ala, Gly, L−Pro, L−Hcy, L−Asp, L−Val, L−Cys, GSH). Reaction conditions: 1.0 mL C−dots (20.0 μg/mL), 0.5 mL OTC (200.0 μM) and 0.5 mL amino acids (1000.0 μM) were incubated in BR buffer (pH = 4.0) at 20.0 °C for 10.0 min. (**D**) Common anions and acids (AA, SO_4^{2-}, NO_3^{-}, Cl^{-}, I^{-}, PO_4^{3-}, CA). Reaction conditions: 1.0 mL C−dots (20.0 μg/mL), 0.5 mL OTC (200.0 μM), and 0.5 mL amino acid (1000.0 μM) were incubated in BR buffer (pH = 4.0) at 20.0 °C for 10.0 min.

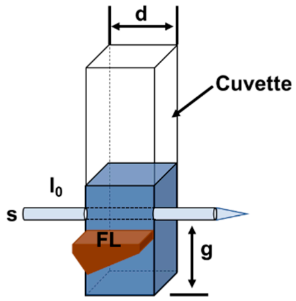

Figure S11. Diagram for examining IFE action in the fluorescence decrease of this biomass-based C−dots at 520 nm. I_0 expressed the excitation beam, FL corresponded to the observed fluorescence beam, s = 0.10 cm stood for the inner diameter of the cuvette, and g = 0.40 cm assigned to the distance of the excitation beam edge to the edge of the cuvette.

According to the characteristics of the cuvette geometry and the absorption in the system of this biomass−based C−dots in BR buffer (pH = 4.0), the corrected fluorescence emission intensity was calculated with the following equation [2]:

$$CF = \frac{F_{\text{corr}}}{F_{obsd}} = \frac{2.3dA_{ex}}{1-10^{-dA_{ex}}} \times 10^{gA_{em}} \times \frac{2.3sA_{em}}{1-10^{-sA_{em}}}$$

In this expression, *CF* expressed the corrected factor; F_{obsd} indicated the observed fluorescence intensity of this biomass–based C–dots at 520 nm, and F_{corr} corresponded to the corrected fluorescence intensity at 520 nm after removing IFE action from F_{obsd}. Besides, A_{em} and A_{ex} assigned to the emission wavelength and the absorbance per centimeter of this biomass-based C–dots, respectively. I_0 indicated the excitation beam, FL expressed the observed fluorescence beam, s = 0.10 cm was the inner diameter of the cuvette, and g = 0.40 cm was the distance of the excitation beam edge to the edge of the cuvette. Considering the obvious difference between E_{obsd} and E_{corr}, the IFE behavior was concluded in the fluorescence decrease of TCs toward this biomass-based C–dots.

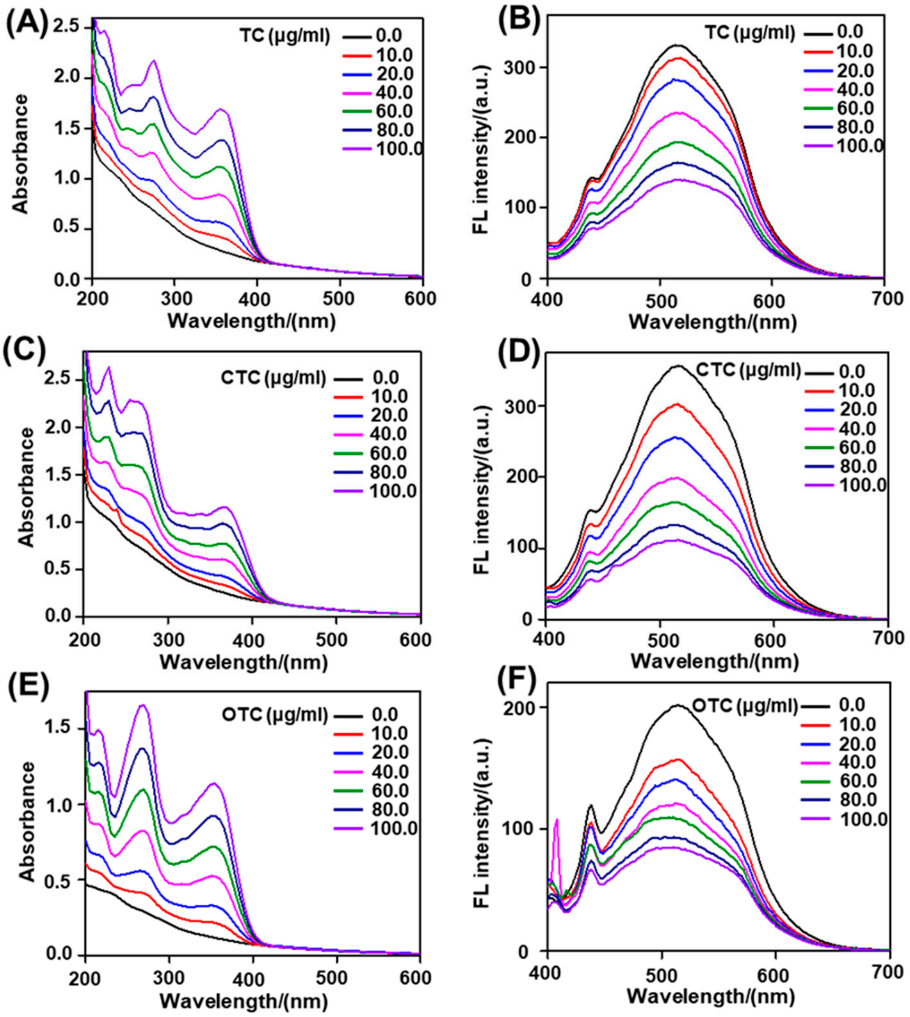

Figure S12. IFE corrected UV−Vis absorption of different concentrations of TCs (**A**) TC. Reaction conditions: 1.5 mL C-dots (50.0 μg/mL) and 1.5 mL TC (50.0 μM) were incubated in BR buffer (pH = 5.0) at 20.0 °C for 10.0 min. (**C**) CTC. Reaction conditions: 1.5 mL C–dots (50.0 μg/mL) and 1.5 mL CTC (50.0 μM) were incubated in BR buffer (pH = 4.0) at 50.0 °C for 10.0 min. (**E**) OTC. Reaction conditions: 1.5 mL C–dots (20.0 μg/mL) and 1.5 mL TC (50.0 μM) were incubated in BR buffer (pH = 4.0) at 20.0 °C for 10.0 min. IFE corrected fluorescence spectra of different concentrations of TCs (**B**) TC. Reaction conditions: 1.5 mL C–dots (50.0 μg/mL) and 1.5 mL TC (50.0 μM) were incubated in BR buffer (pH = 5.0) at 20.0 °C for 10.0 min. (**D**) CTC. Reaction conditions: 1.5 mL C–dots (50.0 μg/mL) and 1.5 mL CTC (50.0 μM) were incubated in BR buffer (pH = 4.0) at 50.0 °C for 10.0 min. (**F**) OTC. Reaction conditions: 1.5 mL C–dots (20.0 μg/mL) and 1.5 mL TC (50.0 μM) were incubated in BR buffer (pH = 4.0) at 20.0 °C for 10.0 min.

Table S2. Exploring the IFE behaviors of TCs on the fluorescence decrease of this biomass−based C−dots at 520 nm.

TCs	C (μM)	A_{ex} (380 nm)	A_{em} (520 nm)	F_{corr}/F_{obsd}	F_{obsd}	F_{cor}	E_{obsd}	E_{cor}
TC	0	0.2179	0.0591	1.3490	330.5	445.9	0.0	0.0s
	10	0.2971	0.0599	1.4652	312.1	457.3	0.05567	0.005752
	20	0.3821	0.6071	1.5978	280.9	448.8	0.1501	0.01124
	40	0.5383	0.0608	1.8543	235.7	437.0	0.2868	0.01973
	60	0.7002	0.0606	2.1390	193.5	413.9	0.4145	0.07167
	80	0.8552	0.0585	2.4267	164.3	398.7	0.5029	0.1058
	100	1.0368	0.0595	2.7901	139.1	388.1	0.5791	0.1295
CTC	0	0.2165	0.0592	1.3469	354.8	477.9	0.0	0.0
	10	0.2867	0.0576	1.4494	298.9	433.2	0.1576	0.09348
	20	0.3685	0.0549	1.5750	252.8	398.1	0.2875	0.1668
	40	0.5258	0.0536	1.8303	196.0	358.7	0.4476	0.2493
	60	0.6724	0.0536	2.0870	161.7	337.4	0.5443	0.2939
	80	0.8596	0.0578	2.4341	131.4	319.8	0.6297	0.3307
	100	1.0252	0.0570	2.7639	110.3	304.8	0.6891	0.3621
OTC	0	0.0893	0.0242	1.1684	199.0	232.5	0.0	0.0
	10	0.1357	0.0227	1.2298	153.7	189.0	0.2276	0.1870
	20	0.1951	0.0283	1.3136	137.2	180.2	0.3106	0.2249
	40	0.2922	0.0232	1.4536	119.4	173.5	0.4	0.2535
	60	0.3914	0.0238	1.6053	106.6	171.1	0.4643	0.2640
	80	0.4953	0.0233	1.7714	92.2	163.3	0.5367	0.29756
	100	0.6009	0.0243	1.9516	83.6	163.3	0.5795	0.2976

References

1. Magde, D., Wong, R., Seybold, P.G. Fluorescence quantum yields and their relation to lifetimes of rhodamine 6G and fluorescein in nine solvents: Improved absolute standards for quantum yields. *Photochemistry and photobiology*, **2002**, *75(4)*, 327–334.
2. Shen, Y.; Wei, Y.; Chen, H.; Wu, Z.; Ye, Y.; Han, D.M. Liposome-encapsulated Aggregation-induced Emission Fluorogen Assisted with Portable Smartphone for Dynamically on-site Imaging of Residual Tetracycline. *Sens. Actuators B Chem.* **2022**, *350*, 130871.
3. Fan, Y.J, Su, M., Shi, Y.E., Liu, X.T., Shen, S.G., Dong, J.X. A ratiometric fluorescent sensor for tetracyclines detection in meat based on pH-dependence of targets with lanthanum-doped carbon dots as probes. *Analytical and Bioanalytical Chemistry*, **2022**, *414(8)*, 2597–2606.
4. Li, C., Yang, W., Zhang, X., Han, Y., Tang, W., Yue, T., Li, Z. A 3D hierarchical dual-metal–organic framework heterostructure up-regulating the pre-concentration effect for ultrasensitive fluorescence detection of tetracycline antibiotics. *Journal of Materials Chemistry C*, **2020**, *8(6)*, 2054–2064.
5. Miao, H.; Wang, Y.; Yang, X. Carbon dots derived from Tobacco for Visually Distinguishing and Detecting Three Kinds of Tetracyclines. *Nanoscale*, **2018**, *10*, 8139–8145.
6. Yang, K., Jia, P., Hou, J., Bu, T., Sun, X., Liu, Y., Wang, L. Innovative dual-emitting ratiometric fluorescence sensor for tetracyclines detection based on boron nitride quantum dots and europium ions. *ACS Sustainable Chemistry & Engineering*, **2020**, *8(46)*, 17185–17193.
7. Hu, X., Zhao, Y., Dong, J., Liu, C., Qi, Y., Fang, G., Wang, S. A strong blue fluorescent nanoprobe based on Mg/N co-doped carbon dots coupled with molecularly imprinted polymer for ultrasensitive and highly selective detection of tetracycline in animal-derived foods. *Sensors and Actuators B: Chemical*, **2021**, *338*, 129809.
8. Li, C., Zeng, C., Chen, Z., Jiang, Y., Yao, H., Yang, Y., Wong, W.T. Luminescent lanthanide metal-organic framework test strip for immediate detection of tetracycline antibiotics in water. *Journal of hazardous materials*, **2020**, *384*, 121498.
9. Li, R., Wang, W., El-Sayed, E.S.M., Su, K., He, P., Yuan, D. Ratiometric fluorescence detection of tetracycline antibiotic based on a polynuclear lanthanide metal–organic framework. *Sensors and Actuators B: Chemical*, **2021**, *330*, 129314.
